# Strategies in Forward Osmosis Membrane Substrate Fabrication and Modification: A Review

**DOI:** 10.3390/membranes10110332

**Published:** 2020-11-07

**Authors:** Nur Diyana Suzaimi, Pei Sean Goh, Ahmad Fauzi Ismail, Stanley Chinedu Mamah, Nik Ahmad Nizam Nik Malek, Jun Wei Lim, Kar Chun Wong, Nidal Hilal

**Affiliations:** 1 Advanced Membrane Technology Research Centre, Faculty of Chemical and Energy Engineering, Universiti Teknologi Malaysia, Johor 81310, Malaysia; diyanasuzaimi@yahoo.com (N.D.S.); peisean@petroleum.utm.my (P.S.G.); afauzi@utm.my (A.F.I.); douglascms@yahoo.com (S.C.M.); karchun@utm.my (K.C.W.); 2 Department of Chemical Engineering, Alex Ekwueme Federal University, Ebonyi State 84001, Nigeria; 3 Department of Biosciences, Faculty of Science, Universiti Teknologi Malaysia, Johor 81310, Malaysia; niknizam@utm.my; 4 Department of Fundamental and Applied Sciences, HICoE-Centre for Biofuel and Biochemical Research, Institute of Self-Sustainable Building, Universiti Teknologi PETRONAS, Seri Iskandar 32610, Perak, Malaysia; junwei.lim@utp.edu.my; 5 NYUAD Water Research Center, New York University Abu Dhabi, Abu Dhabi 129188, UAE

**Keywords:** thin film composite, FO substrate, internal concentration polarization, fouling mitigation, fabrication and modification, electrospinning, blending, template-assisted technique, surface coating

## Abstract

Forward osmosis (FO) has been recognized as the preferred alternative membrane-based separation technology for conventional water treatment technologies due to its high energy efficiency and promising separation performances. FO has been widely explored in the fields of wastewater treatment, desalination, food industry and bio-products, and energy generation. The substrate of the typically used FO thin film composite membranes serves as a support for selective layer formation and can significantly affect the structural and physicochemical properties of the resultant selective layer. This signifies the importance of substrate exploration to fine-tune proper fabrication and modification in obtaining optimized substrate structure with regards to thickness, tortuosity, and porosity on the two sides. The ultimate goal of substrate modification is to obtain a thin and highly selective membrane with enhanced hydrophilicity, antifouling propensity, as well as long duration stability. This review focuses on the various strategies used for FO membrane substrate fabrication and modification. An overview of FO membranes is first presented. The extant strategies applied in FO membrane substrate fabrications and modifications in addition to efforts made to mitigate membrane fouling are extensively reviewed. Lastly, the future perspective regarding the strategies on different FO substrate layers in water treatment are highlighted.

## 1. Introduction

In many parts of the world, rapid human development and economic growth combined with worsening climate change are creating negative pressures on water demands [1,2]. The limited access of clean water in some arid areas has resulted in long-term ecosystem damage and has threatened human security through waterborne-related diseases. These overarching global crises take priority over everything, further pushing researchers towards developing innovative, advanced, and affordable water treatment technologies to address the challenges. As the world continues to navigate the pandemic of coronavirus diseases 2019 (COVID-19), now more than ever the access to clean and safe water is critical to maintain a healthy lifestyle. Improving water and wastewater treatment systems is especially important in ensuring continual supply of clean and safe water for ourselves, our families, and our surroundings. Amidst the global water scarcity, various water/wastewater treatment technologies such as solvent extraction, membrane filtration [3,4,5], adsorption [6,7,8,9], chlorination [10,11], and electrocoagulation– flocculation [12,13,14] have been developed. The implementation of water reclamations through these technologies imposes a positive impact on treating water and decreasing health complications. Among the existing technologies, membrane-based separation has steadily gained increasing acceptance over the years and has often become the first choice for a reliable performance. Principally, membrane-based separations involve the selective filtration of solutes through pores of different sizes while allowing only water molecules to pass through. Membrane technology is crowned by sustainability criteria in terms of handy design programs, an easy scaling up process, minimal environmental impact, flexibility, and adaptability [15,16,17]. In addition to this, helpful literature and improved knowledge have led to this technology becoming much more familiar. Membrane technology has started to become a favorable treatment process since the development of cellulosic reverse osmosis (RO) membrane via phase inversion developed by Loeb and Sourirajan in the 1960s [18]. This innovation has reached maturity and has grown in line with the increasing acceptance of membrane-based wastewater treatment and desalination technology to deal with increasing water demands and stringent regulations [19,20]. The engineered applications of the four most common pressure-driven membrane processes, i.e., RO, nanofiltration (NF), ultrafiltration (UF), and microfiltration (MF) include water desalination, reclamation, purification, and wastewater recycling.

In particular, RO, with the finest degree of separation, has achieved huge commercial success due to its high efficiency in removing dissolved ions, particles, and bacteria in the water, as well as having broad tolerance with feed stream of different qualities. The operation of RO requires high hydraulic pressure as a driving force. This has imparted negative consequences to the cost, energy consumption, and fouling propensity of the processes. As a strategy to address these limitations, attention has been switched to the application of osmotically driven processes as an alternative technology for desalination and wastewater treatment. Forward osmosis (FO), the most common osmotically driven membrane process, stands out as the most promising alternative for RO processes due to its inherently low fouling tendency, easier fouling removal, and energy efficiency when compared to pressure-driven–type membrane processes [16,17,18,19]. Owing to these attractive inherent features, FO has been used as a concentration and dilution process in diverse areas including food processing [21,22,23,24], wastewater treatment [25,26,27], desalination [28,29,30,31], and power generation [3,32]. In medical application, FO assists in controlling the release of drugs with low solubility [33,34] and in preserving properties of feed (nutrition, taste, quality, etc.) when it comes to the pharmaceutical industry [35]. FO is also known as an ideal pretreatment step in many integrated membrane processes for desalination/wastewater treatment [36,37,38]. However, the high potential shown by the FO process to unravel the present-day water shortage has been hindered to some extent by some practical challenges. The frequently encountered issues of FO include concentration polarization (CP), fouling, weak membrane mechanical strength, low membrane flux, and high expense to regenerate draw solution and recover water from draw solution, which is behind the unfavorable separation performance of the FO membrane [39]. These factors have triggered more developmental and implementation research into a sustainable FO membrane separation process for a proper acknowledgement and understanding of the FO process. Various improvement attempts have been made to further advance FO technology in order to seize the foothold in mainstream water/wastewater industries.

The dominant factors affecting FO performance along with potential implications on the overall process have been investigated in numerous studies [40,41,42,43]. Some of the important factors are membrane properties, operating conditions, and types of draw and feed solutions [44]. Many studies have mentioned the crucial role of membrane properties such as charge, roughness, and pore size on the transport behavior and hence the overall FO performance [43,45,46,47,48]. An ideal draw solution should be capable of generating high osmotic pressure, reducing reverse diffusion, and easily re-concentrating and recovering in order to enhance driving force for efficient separation and water transport [49,50]. Low molecular weight salts, especially NaCl, are widely applied as draw solution due to their high solubility and re-concentration simplicity. It is also notable that characteristics and properties of draw solution have profound effects on the degree of membrane fouling as well as water flux [51]. In addition, operating conditions including solution chemistry (pH and temperature) and membrane orientations can be altered to reduce the effects of fouling. Membrane surface charge varies with pH of feed solution, where high charge facilitates diffusion of the draw solutes in the substrate [52]. Meanwhile, osmotic pressure, fluid viscosity, mass transfer, and solubility are dependent on solution temperature. It is essential to keep solution temperature constant so that the membrane performance is not altered [53]. Zhao and Zou [54] observed that water and salt permeabilities increased at a higher temperature as viscosity decreased and water diffusivity increased. In FO operations, membrane can be oriented to FO mode or AL-FS (active layer facing the feed solution), which provides a more stable and higher water flux than that in the alternative membrane orientation, i.e., pressure-retarded osmosis (PRO) mode or AL-DS (active layer facing the draw solution). Practically, the AL-FS orientation is more favorable when the membrane is employed in wastewater treatment since AL-DS orientation would lead to more severe and irreversible membrane fouling [55].

A large step in heightening the performance of the FO process is through the upgrading of the FO membrane. Unceasing pursuit of fabrication strategies for low CP and antifouling membrane remains a major theme in terms of this topic. Thin film composite (TFC) membranes are currently popularly used membranes owing to their superior performances in terms of water flux and salt rejection [56]. TFC membrane is composed of a substrate layer and a selective layer on top of the substrate [57,58]. The layers are fabricated and tailored separately towards each optimum structure and property. The produced membrane can be with or without the thin nonwoven layer underneath the substrate layer. To date, numerous studies related to TFC-FO membranes have been devoted to polyamide (PA) active layer and substrate layer modification to combat membrane fouling, alleviate CP, and achieve high flux performance [59,60,61,62,63]. Previous investigations revealed that surface roughness, porosity, pore size, and hydrophilicity of the substrate have substantial influence on the microstructure of the PA layer formed and the membrane performance [16,56,64]. The high porosity and low tortuosity membrane collectively lower the CP phenomena, especially internal concentration polarization (ICP). This has hence attracted researchers to fabricate and modify the substrate of the FO membrane to transform it into a valuable finished membrane. For instance, current studies are focusing on the selection of additives with a proper modification technique as they affect substrate characteristics and eventually determine the ultimate performance in the FO process [65]. Introduction of a suitable additive/nanofiller to the casting solution would enhance pore interconnectivity and/or hydrophilicity [66]. Many endeavors have been made by worldwide researchers on the use of additives, including hydrophilic polymer, nanomaterials, pore former via blending, surface coating, template-assisted, and electrospinning, all being among the most popular fabrication routes that promote construction of desired substrate properties [65].

The advancement has been rapidly made in the preparation and modification of FO membranes all the while. There is a need to review these developments in order to pave the way towards further studies in the future. Recently, Akther et al. [67] provided a state-of-the-art summary of the nanomaterial-modified PA layer, substrate layer, and the surface of the PA TFC-FO membranes. Suwaileh and colleagues [68] presented a review on the advancement of synthetic polymer and the substrate, focusing on the fabrication and chemical modifications. Aquaporin-based biomimetic FO membranes, which are different in fabrication technique and behavior, were also included. Their reported progress, however, is limited to research studies until 2017. Meanwhile, a comprehensive review by Goh et al. [69] outlined up-to-date strategies used in membrane designs and fabrications, also highlighting fouling mitigating strategies, particularly for wastewater treatment. However, the progress of substrate fabrication and modification to deal with ICP and fouling specifically for FO desalination and wastewater treatment based on more recent progress has not been reviewed.

This article postulates a review of the developments in FO nanocomposite membranes within these five years by focusing on the tailoring of the substrate layer to improve the characteristics for the applications of wastewater treatment and desalination The key part of this review is an in-depth discussion of the substrate layer for polymeric membrane in minimizing the adverse effects of ICP and membrane fouling, thereby enhancing membrane performance. We briefly describe the FO principle and membrane. The main challenges confronted by the substrate of FO membrane are also discussed. The discussion is followed by motivation for membrane modification and reviewing the available approaches to enhance the performance, assorted into four main categories, including electrospinning fabrication, blending, templated-assisted, and surface coating. The corresponding approaches are reviewed with examples from the literature, emphasizing their advances and challenges with respect to improving FO membranes in the future. Finally, a conclusion and perspective are stated according to the reviewed progress to date. These perspectives provide researchers with a useful reference to further accelerate progress in substrate membrane development towards a more sustainable and successful FO membrane operation.

## 2. Overview of Forward Osmosis Membranes

### 2.1. Forward Osmosis Membrane

Like for other membrane processes, the high-performance semipermeable membrane is the key to achieving a highly efficient FO process. Technically, a membrane that is comprised of dense, non-porous, and selectively permeable materials can be used for the FO process. On the basis of their fabrication materials, these available membranes would be categorized as cellulosic membranes, thin film composite (TFC) membranes, and chemically modified membranes [1]. Polymeric membranes account for the largest proportion of the currently installed membranes. In a period of history, typical cellulose acetate (CA)-RO membranes and TFC-RO membranes were feasibly explored for the FO process, as no membrane was designed specifically for the process. CA membrane profits from its comparative hydrophilicity, good mechanical strength, and wide availability balanced with cost-effectiveness [70,71]. This cellulosic membrane is commonly applied for nonsolvent-induced phase separation methods for both active and substrate layers, which is relatively easy to scale up and has good hydrophilicity and mechanical strength [58]. Despite favorable characteristics of CA membranes, coordinating trade-off between water permeability and rejection is challenging due to pH sensitivity, temperature resistance, and biofouling mitigation [39,72]. During the FO process, cellulosic membranes are usually susceptible to chemical hydrolysis, low selectivity, and biological attack [14]. Although innovation has been implemented on the CA-FO membrane, some its membrane performances are generally inferior to the TFC membrane, which is primarily correlated to the structural compaction and high operating pressures [57,73,74,75]. Thus, continuing research is needed on the TFC-FO membrane for the FO process because of its porous substrate and thin active layer that make it capable of promoting high water permeability and selectivity, respectively.

Later, Hydration Technologies, Inc. (HTI) developed the first generation of commercial FO membranes, one of which has a characteristic structure of cellulose triacetate (CTA) embedded with thin polyester mesh support. The flux of the TFC-FO spiral element was twice of that existing CTA membranes [76]. This achievement has provided a new benchmark in the development of FO membranes. Nevertheless, developing a FO membrane with superior water permeability and salt rejection is still one of the biggest challenges for the practical application of FO. The TFC-FO membranes have been designed differently from the TFC-RO membranes, especially with regard to the substrate (polymeric support layer), which for FO membranes is considerably more porous, more hydrophilic, and thinner. The most common polymers used for the preparation of TFC-FO membrane substrate are polysulfone (PSf), polyethersulfone (PES), polyacrylonitrile (PAN), polyvinylidene fluoride (PVDF), and cellulose derivatives. TFC membranes are the predominant kind of membranes as of now, thanks to their flexibility in design, as both the active and substrate layers can be tailored for specific needs [77]. Moreover, they provide higher selectivity and productivity with less energy consumption as compared to typical asymmetric membranes [47]. The topmost active layer is designed as a barrier that blocks feed solutes or contaminants while allowing only water molecules to be permitted. Differently, the substrate serves as the foundation of the composite membrane, providing mechanical strength and flow pathway. Beyond the basics, it also lays a versatile platform for growth of the PA layer. This membrane structure imposes less resistance to mass transport and improves the overall membrane productivity. The transport of components, namely, water and solute(s), depends on several parameters such as water permeability (A), solute permeability (B), and the structural parameter of the substrate layer (*S*), which are intrinsic membrane parameters [47].

As generally recognized, the fouling behavior in the FO processes has a strong dependence on the membrane-selective layer, while ICP is an inevitable key issue in the substrate layer causing a reduction in water flux [56,78,79]. As such, improvement of FO semi-permeable membranes necessitates further research and new thoughts in order to achieve great performance. On the basis of the data in the literature, the desired criteria of ideal membranes for FO would be (1) a dense and ultra-thin active layer for high solute rejection; (2) having a porous, thin, and low-tortuosity substrate to minimize ICP effects, thereby increasing flux and reducing membrane fouling; (3) hydrophilic substrate increasing the wetting of small pores for high flux and low fouling propensity; (4) and a robust mechanical, chemical, and thermal stability substrate to sustain both long-term operation and hydraulic pressure [16,80,81]. Therefore, alongside production of the commercial HTI-TFC membrane, there has been a rise in the attempt to modify and fabricate TFC membranes with essential properties suitable for FO applications. A wide range of TFC membranes have been implemented for the FO process in both flat sheet and hollow fiber configurations. In parallel to the advances of TFC flat sheet [40,61,82,83,84,85], hollow fiber membranes have been widely fabricated for FO application, owing to their self-supported structure and high packing density [86,87,88,89,90]. Membranes are usually provided as flat sheets for water treatment applications dealing with high concentration of fouling agents or solutions with high viscosities [91]. Meanwhile, large-volume water treatment commonly applies hollow fiber FO membranes [86,92,93].

The non-solvent-induced phase separation method is commonly used for substrate fabrication due to its time-effective, cost-efficient, and straightforward preparation technique [94]. Throughout the method, a homogeneous polymer solution is immersed in a coagulation bath, hence transforming a liquid into a solid state in a controlled manner. Preparation of asymmetric phase-inversion membranes can be done through different procedures: non-solvent-induced phase separation, thermal-induced phase separation, evaporation-induced phase separation, and vapor-induced phase separation, as long as they are soluble in an appropriate solvent [94]. However, above all, phase inversion via non-solvent-induced phase separation is the most widely used technique for casting polymeric membranes. Subsequently, membrane pores are formed from the liquid phase, which is poor in polymer and surrounded by the solid phase rich in polymer, where the porous structure straightforwardly corresponds to the flux rate [45,95]. To attain a good FO performance, the perfect choice of solvent mixture, polymer concentration, as well as some parameters (temperature, air humidity, additives, and coagulation bath) are important factors for the formation of desired substrate.

### 2.2. Challenges Confronted by FO Membrane

Although facing some critical issues that limit the applications, research on FO membranes is still being conducted, even though the osmotically driven membrane processes have been extensively investigated with some noticeable results being reported. As mentioned earlier, ICP and fouling are the major hiccups in terms of the development of a TFC-FO membrane as they restrict the overall performance [57].

#### 2.2.1. Concentration Polarization

**Figure 1:**
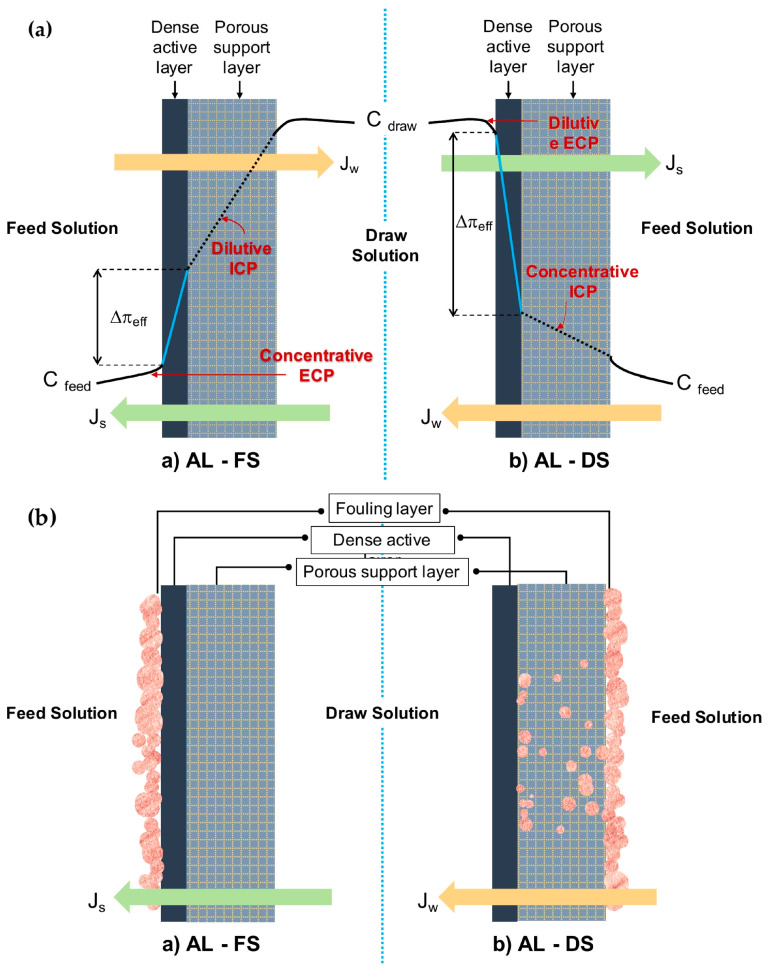
A conceptual illustration of the (**a**) membrane orientations AL-FS (active layer facing the feed) and AL-DS (substrate layer facing the feed) with a concentration polarization (CP) profile and (**b**) membrane fouling in forward osmosis (FO) membrane at different orientations.

Concerted research efforts have been dedicated in terms of better understanding and mitigating polarization effects imposed on all asymmetric membranes—pressure-driven and osmotically driven. The polarization issue relates especially to ICP, which occurs inside the pores of the porous substrate, remaining as a main constraint in FO. In FO, the differential osmotic pressure and solvent flow has been effectively reduced as feed solution is more concentrated on one side of the membrane and the draw solution is more diluted at the other. An unsatisfying value of water flux and reverse salt flux is obtained due to the severe mass transfer resistance built up both inside and around membranes during the osmosis process (CP phenomena) [50,96,97]. The magnitude of the effects depends on the membrane nature and mode of orientation, as illustrated in Figure 1a. Conceptually, CP falls into two main categories that occur concurrently, i.e., external concentration polarization (ECP) and ICP [73]. The orientation of AL-FS giving rise to concentrative ECP and dilutive ICP simultaneously. AL-DS on the other hand, would experience dilutive ECP and concentrative ICP.

ECP occurs outside of membrane due to solute accumulation within the surface of the dense active layer, which could possibly be counteracted by optimizing flow conditions and hydrodynamics design [98]. Another implemented solution to this problem is topping the porous substrate with a highly selective active layer [99,100]. Meanwhile, the difficult solute diffusion through the porous substrates mainly results in serious ICP. The occurrence is induced by the thick substrate layer and high *S* value that contributes to a massive decline in the effective osmotic driving force and thus the flux. [73]. The ICP problem is always more pronounced and burdensome than ECP [100]. ICP residing in the membrane structure cannot be easily controlled, neither by stirring nor spacer design [98]. Therefore, the key to solve this problem is to construct the substrate with its interior pores highly interconnected by understanding that the mechanism on ICP is essential in order to innovate membrane design and synthesis. The breakthrough for FO came with the innovation of tailored FO membranes, generating higher fluxes to ascertain the adverse effect of ICP [63,73]. For instance, it is known that a hydrophilic substrate with a smaller membrane *S* parameter are effective against ICP. The hydrophilic substrate allows a complete wetting throughout the structure. A hydrophilic substrate with improved wettability is beneficial with regards to water, and solute molecules facilitate transportation, decrease the effective tortuosity, and increase the porosity to reduce the air entrapping in the membrane pores. A combination of the effects led to remission of ICP and improvement of the performance while maintaining strength or flexibility of the membrane.

An insight to the degree of ICP is quantified using the *S* parameter and is primarily influenced by three intrinsic properties of substrate: wall thickness, porosity, and tortuosity, as defined in Equation (1):

(1) $$\;\;\;\;\;\;\;\;\;\;\;\;\;\;\;\;\;\;\;\;\;\;\;\;\;\;\;\;\;\;\;\;\;\;\;\;\;\;\;\;\;\;\;\;S=\frac{t\tau }{\varepsilon }\;\;\;\;\;\;\;\;\;\;\;\;\;\;\;\;\;\;\;\;\;\;\;\;\;\;\;\;\;\;\;\;\;\;\;\;\;\;\;\;\;\;\;\;\;\;\;\;\;\;\;\;\;\;\;$$

where *t* is the thickness, τ is the tortuosity, and ε is the porosity of the substrate layer. The *S* parameter is inversely proportional to membrane porosity. Less air entrapped within the membrane pores would increase the porosity, make the flow path less tortuous, and provide a direct path from draw solution to the PA layer. These structural characteristics reduce the value of the *S* parameter to increase effective osmotic difference across the membrane. The swift transport of water and solute molecules together with smaller value of *S* parameter contributes to IC suppression and higher water flux [56,73]. However, the value of the *S* parameter can never be lower than the wall thickness of the substrate layer. Analysis has shown that the thickness of substrate affects the *S* parameter by a factor of 10 compared to porosity and tortuosity, as their values only change within a limited range [101]. Membranes that suffer from *S* value that is too high would possibly produce high permeance but not the fluxes. Only at lower *S* values can the membrane permeance substantially contribute to the higher water flux. Other substrate parameters to be considered besides those three are the pore size and morphology hydrophilicity, and charge. To effectively implement this platform technology, the substrate structure needs to be engineered to achieve minimal thickness and tortuosity, as well as high porosity and hydrophilicity, without the mechanical fragility of the latter.

#### 2.2.2. Membrane Fouling

Besides ICP, FO membranes also suffer from fouling, a long-standing problem shared by other membrane processes [1,102,103,104]. Fouling arises from a variety of factors associated with surface chemistry, membrane morphology, and structural properties. In general, solute particles that accumulate or adsorb either on the surface of a membrane or are entrapped within its pores causes a fouling [105]. Application of hydrophobic polymers i.e., PES, PVDF, PSf, and polypropylene (PP) used to fabricate the membrane do not swell in water, but they are likely to adsorb foulants. Physical and chemical interaction of foulants and the membrane results in poor membrane productivity by reducing the quality and quantity of permeate, i.e., pure water fluxes, as well as shortening membrane lifespan depending on how pronounced it is [55,106]. After comparing the fouling of FO with RO, Holloway et al. reported that the flux decline rate was greater with RO [107]. The authors speculated that both the lower extent of FO fouling and its reversibility (enabling easy cleaning) was due to the effects of hydraulic pressure upon the foulants on the membrane surface, which occurs rapidly in RO [69,107]. Still, a membrane with a long-term antifouling ability needs to be developed so as to have the prominent advantage of keeping flux decline caused by fouling to a minimum extent. Through this way, the FO membrane could be highly known for efficiently treating fouling/saline wastewater and thus outmaneuvering pressure-driven salt-rejecting membranes such as RO and NF membranes.

Like the fouling phenomenon in pressure-driven membranes, fouling for the FO membrane also experiences different types of fouling, namely, biofouling, colloidal fouling, inorganic scaling, and organic fouling [1]. Biofouling is a complex form that results from adhered microorganisms (from feed) on the membrane surface that subsequently form a biofilm, increasing its resistance to water [108,109]. Meanwhile the deposition of suspended particles (e.g., clay, silica) and organic matter (e.g., humic substances, proteins, and amino acids) on the membranes develop other types of fouling called colloidal and organic fouling, respectively [69]. Colloidal fouling on the surface leads to less porous fouling layers, hence reducing the flux. Scaling arises from the high concentration of some inorganic ions such as metal sulfates and carbonates in the water, leading to precipitation near/on the membrane surface [110]. In real-application conditions, different types of foulants almost always coexist in natural waters, resulting in simultaneous occurrence of different types of fouling that can influence each other. It is essential to determine the degree and nature of the fouling so as to select the appropriate cleaning and modification strategy.

As schematically diagrammed in Figure 1b, fouling in FO tends to occur internally and externally, at either on the active layer, on the surface, or inside the substrate layer, which is dependent on the type of operation modes. The literature claims that external fouling occurs in AL-FS mode as the deposition of foulants take place on the active layer, forming a cake-type layer. Surface properties such as surface roughness have a greater effect on this type of fouling than other properties (e.g., surface hydrophilicity), therefore enabling easier removal and cleaning. Differently, under AL-DS mode, the constriction of pores due to the deposited foulants on the active layer that is trapped within the membrane leads to internal fouling, which is very hard to clean up [31,80]. The fouling was dominated over structural properties of the support layer, which is more intense compared to AL-FS mode. Additionally, entrapment of foulants in the support layer would reduce porosity and enhance the effects of ICP in membrane, degrading the performance with a consequent increase of energy and membrane replacement costs.

## 3. Motivations of FO Membrane Modification

Despite the fact that ICP and fouling cannot be completely avoided in the osmosis process, upgrading of the properties of surface and structural properties of substrate is an appreciable perspective that ought to be considered in order to achieve excellent substrate properties without compromising flux performance. Recent literature has shown that modification focused on the substrate layer is much less studied in comparison with the selective active layer. For this reason, ongoing experiments and modeling on substrate construction are increasing in order to tackle afforested issues and meet the requirements of practical applications. On the basis of the analysis, it is clear that the porous nature (pore size, size distribution, and porosity) and the surface properties (hydrophilicity and roughness) of the substrate layer significantly affects the crosslinking degree during interfacial polymerization and correspondingly the thickness and morphology of the PA layers formed on top of the substrates [58]. This consequently leads to variation of the performances of the FO membranes including water permeability, salt rejection, and fouling resistance.

It would be of benefit to produce a promising TFC-FO membrane where the substrate layer adequately supports the active layer during both formation and operation. Another important aspect is long-term chemical and mechanical stability, as well as an efficient route for large-scale fabrication. It was found that the effect of ICP can be alleviated by minimizing the *S* value of the membrane substrates. The *S* parameter proportionally decreases as the porosity increases, while increasing with the thickness and tortuosity of the substrates. Such characteristics increase the mass transfer and reduce the ICP. As a main step in controlling ICP, the substrate layer hence needs to be resigned to incorporate a combination of characteristics: thinness, high porosity, excellent hydrophilicity, and less pore tortuosity to minimize ICP and maximize water flux in FO. Similarly, considerable efforts have been made in PA layer modification in recent years as an improvement in the aspects related to antifouling and anti-biofouling properties. However, as substrate has a synergic effect on the active layer, substrate modifications are needed so that the membrane has lower tendencies to fouling and so the performance will not deplete rapidly; hence, small maintenance is needed.

Electrospinning as a fabrication approach as well as modification of substrate surface and substrate matrix through several techniques such as blending, surface coating, template-assisted, layer-by-layer assembly, and double-skinned membrane are among the strategies necessary to boost FO membranes. Modification of TFC-FO membranes, i.e., surface and bulk modification with advanced nanostructure materials, would generate feasible platforms and collective properties, as well as marked performance improvement [111]. Incorporating unique characteristics of nanoparticles, ionic groups, and hydrophilic functional groups such as hydroxyl, amine, carboxyl, and sulfone into the polymer matrix are all conducive strategies to enhance water flux, antifouling capacity, and overall compatibility. As surface roughness and charges also affect the membrane property, coatings have become an appropriate option. The surface coating with a nonporous dense layer would also block the foulants from going through the skin layer, thus avoiding internal fouling. Regarding the roughness of the substrate membrane, the smooth surface tends to weaken the skin layer–substrate interaction. In order to tailor the surface roughness, effective modification strategies need to be established in the future exploration of FO membranes.

## 4. Overview of Fabrication and Modification Techniques

**Figure 2:**
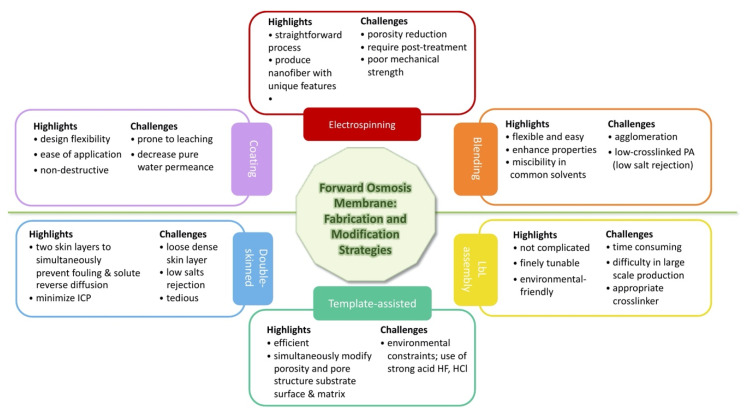
Summary of the pro and cons of substrate modifications of on polyamide thin film composite (TFC) membranes.

**Figure 3:**
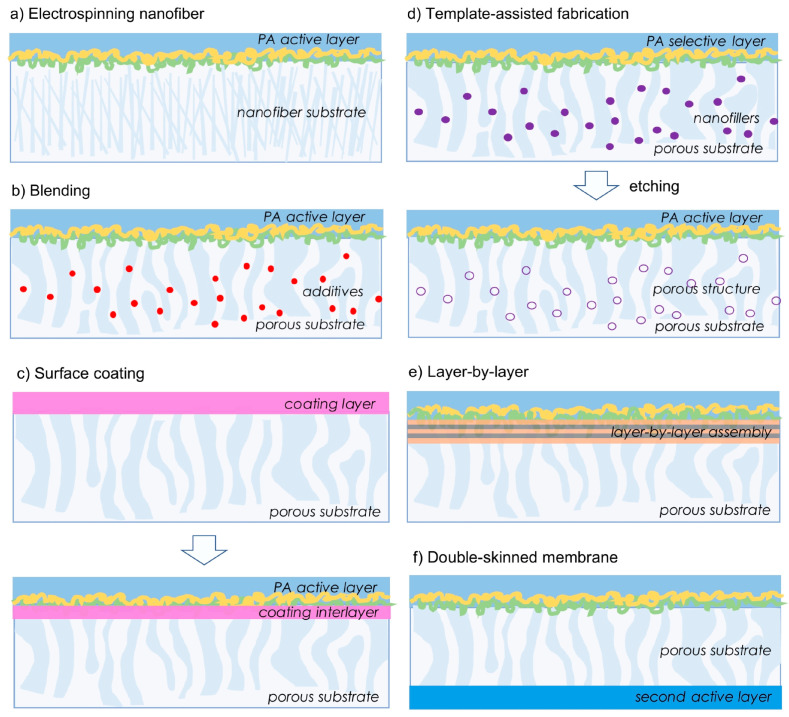
Schematic illustration representing strategies for substrate fabrication and modification; (**a**) electrospinning nanofiber; (**b**) blending technique; (**c**) surface coating; (**d**) template-assisted fabrication; (**e**) layer-by-layer assembly; (**f**) double-skinned membrane.

In this review, close attention is paid to the role of the membrane substrate layer in terms of providing exciting avenues for FO in desalination and water reclamation processes. The application of asymmetric membranes in osmotically driven membrane processes require effective properties in terms of porosity, thickness, hydrophilicity, and surface charge in order to be able to improve its performance. As reported, high hydrophilicity and fully wet substrate allow for effective water transport, otherwise the trapped vapor or air may further block the water flux, reducing the effective porosity and dramatically exacerbating ICP [78]. Fabrication via electrospinning, modification through bulk modification, or surface modification are usually applied to the substrate to increase hydrophilicity, reduce thickness, and adjust porosity [58]. This could be achieved through various methods, particularly by incorporating additives/nanofillers via plasma treatment, grafting, blending, and coating [58,112], or redesigning the FO membrane structure, e.g., double-skinned membrane using layer-by-layer (LbL) assembly [113]. Figure 2 provides a pros and cons summary of the substrate fabrication and modification that is based on the studies reviewed in this contribution. Meanwhile, a schematic illustration representing the strategies is demonstrated in Figure 3.

### 4.1. Electrospinning Nanofiber

Most prior studies focused on fabricated TFC-FO membrane substrates through the phase inversion method for finger-like or needle-like pores to increase the mass transfer within the structure. Still, the poor pore connectivity in the substrates leads to relatively high tortuosity, thus potentially increasing the *S* value [114]. Substrates with minimal tortuosity and thickness, and high porosity coupled with open interconnected pore and high surface area can be accomplished by replacing the substrate casted with an electrospun nanofiber substrate (nanoscale fiber diameters) for a tremendous FO achievement [51,115,116]. Since realizing its potential for water purification, the electrospinning technique has been widely used for polymeric membrane fabrication. The operating conditions and solution parameters during electrospinning determine the morphology of the resulting membranes [39,117]. A variety of polymeric materials can be used for electrospinning, among them, PSf, PES, PVDF, and PAN [83]. The flexibility in material selection and structure manipulation of electrospinning enables the researcher to design and construct nanostructured membranes with desired characteristics to deal with ICP. In previous reported studies, electrospun nanofiber-supported TFC-FO membranes exhibited smaller *S* and excellent water flux, owing to the porous and less tortuous nanofibrous substrate layer [43,51,118,119]. In spite of these, electrospun membranes often end up with post-treatment such as thermal treatment to add functionality or to improve intrinsic membrane properties, e.g., pore size distribution, or mechanical or thermal properties [119]. The necessary post-treatment stage leads to porosity reduction and membrane integrity, which demands further improvement.

### 4.2. Blending

Blending is by far the simplest and most efficient method to change the chemical and mechanical functionalities of the membrane [120], but for polymers, it has limited applicability, which is principally attributed to their limited miscibility of hydrophobic and hydrophilic polymers. Polymer blend is a process where two organic polymers blend in a homogeneous dope solution in the presence of a solvent and/or an additive under mild conditions [66,121,122]. It is conducted to obtain a membrane with desirable morphologies and performance by controlling both the thermodynamics of the casting solution and the kinetics of the demixing process. Generally, hydrophilic–hydrophobic blend polymers such as sulfonated PES and PES substrate render better fluxes and fouling tolerance in comparison to the pristine membrane [123]. Membrane structure, pore tortuosity, porosity, and thickness can also be altered by blending in pore forming additives such as polyethylene glycol (PEG), polyvinylpyrrolidone (PVP), and LiCl [66,71,95,124,125].

In parallel to the development of TFN-FO membranes, prior findings have shown that incorporating nanomaterials into substrate have spawned noticeable improvements in permeability, water flux, salt rejection, and antifouling behavior [121,122,126,127]. The growing development of nanotechnology has brought considerable improvement to conventional polymeric membranes. Converging nanomaterials as nanofillers, i.e., carbon-based nanomaterials, metal oxides, and nanocomposite materials on membrane science, has become the leading research interest, not only for conventional pressure-driven membranes but also for emerging FO membranes [82,128,129]. In comparison with hydrophilic polymer and pore former as substrate modifiers, nanomaterials can flexibly promote construction of favorable substrate properties in multiple ways. This includes improving the solvent exchange rate of the solvent and the nonsolvent, surface chemistry (wettability, hydrophilicity), microstructure (pore inter-connectivity, porosity), and mechanical stability of the substrates to increase rejection efficiencies and water permeability [94,122]. Introducing nanofillers into the TFC substrates through direct physical blending is the most commonly used way. The pristine or modified nanofillers can be embedded into the polymer matrix, whether by blending them in a coagulation bath or in the polymer solution [67]. Nevertheless, most reported studies preferred blending nanofillers in a polymer solution to other techniques [130,131,132,133,134]. The addition and dispersion of nanofillers directly into the substrate layer not only leads to alteration properties of the substrate itself, but also brings significant effects on the polyamide formation [128]. Importantly, for the blending technique, aspects such as the appropriate amount of additives, specific functional groups, as well as the uniform dispersion, are prerequisite to ensure their compatibility and effective blending.

### 4.3. Template-Assisted Fabrication

A very substantial research has been accomplished to assess the influence of TFC nanocomposite substrate on the performance attributes of the FO system [82,135,136,137,138,139]. Past studies have witnessed that some of nanofillers are prone to agglomeration due to their high aspect ratio and poor compatibility with the polymer matrix, leaching, and toxicity, which place a limit on the effectiveness of substrate through nanofiller addition, despite the approach being proven convenient [127,140]. Therefore, further exploration on the methodology and on advanced nanofillers are necessary to develop a novel membrane to break the trade-off between membrane filtration resistance and obtained good water quality. To address this issue, the template-assisted method, also known as the pore-forming method, has been proposed to in order construct porous networks within the substrate. Nanofillers work as a template for pore formation and connectivity in the substrate that are eventually removed, followed by template etching and cleaning [141]. Templating materials can be easily formed using nanofillers to obtain porous substrate with different pore structures. The nano porous structure of nanomaterials added into dope solution can induce a fast solidification process, resulting in the formation of more pores and a thinner membrane [142]. Researchers have assumed that pores of the porous substrate are the filter channels used to speed up flow of the feed or draw solution within the membrane pores if the tortuosity factor is reduced [141,143]. The more pores there are in the substrate surface, which is attached with and underneath the PA layer of the TFC-type FO membrane, the more effective the area of the FO process. By this strategy, better separation flux could be achieved due to the changes in the physical structure of the membrane.

### 4.4. Surface Coating

Apart from blending technique, surface modification realized via coating is another approach to introduce nanofillers to the membrane. Coating is another modification technique that can uniformly hydrophilize the substrate and/or impart good fouling resistance properties [144]. Coating a thin hydrophilic layer of various nanoparticles on the membrane surface via crosslinking, dip-coating, spray-coating, vacuum filtration, or physical adsorption offers high-level membrane performance [96,145]. As a result, the membrane surface is covered by a coated layer, either on top of membrane surface or interlayer between PA and the substrate layer (illustrated in Figure 3c. It is worth noting that the interlayer could lead to high hydrophilicity of inner pores associated with enhanced stability, small pore size distribution, and fine surface. It is possible that the coating technique could be used to produce layer-by-layer membrane or double-skinned/sandwiched membrane. There is a possibility of penetration of the coated layer into the pores of the membrane, and thus high molecular weight polymers should be used for the coated layer. Polydopamine (PDA) has been proposed as a promising material for surface coating [60,144,146,147]. The versatility of PDA coating attributed to its great adhesion to any substrates and natural hydrophilicity PDA coating has attracted the attention of many researchers [144]. However, PDA is prone to degradation by strong oxidizers or extreme pH. Such long immersion time in a PDA solution causes a thicker coating layer on the membrane surface, which leads to pore blockage and results in the decrease of flux of the membrane.

Another useful and practical fashion to form the rejection skin on a porous substrate for fabrication of high-performance TFC membrane is via the LbL technique, which can be performed under a mild preparation environment [148]. This technique involves the deposition of polycation and polyanion on a porous membrane, alternatively forming thin and compact layers tightly attached to the substrate surface as a result of electrostatic interactions. Tunable assemblies of multilayers can also be achieved by hydrogen bonding, hydrophobic interactions, and Van der Waals forces, which influence the stability, morphology, and layer thickness of the film formed [129]. Importantly, appropriate chemical crosslinking strategies are core to forming a high compatibility LbL membrane. The LbL assembly has been utilized for membrane fabrication to relieve ICP effect and endow better antifouling performance. Interestingly, LbL is one of the possible approaches to fabricate double-skinned membrane. Employing the technique, the first systematic study on double-skinned LbL FO membranes was prepared by Qi et al. [148]. The membranes demonstrated excellent FO water flux, low specific reverse solute diffusion, and superior anti-fouling ability. Rejection skin on the feed solution side played a pivotal role in reducing the membrane fouling propensity. Such good results enable the LbL-based membrane to be applied for application in settings such as concentrating feed or product recovery from the feed. However, this membrane may not be suitable for seawater desalination applications, due to its relatively low retention against salt. To advance LbL FO membranes for real applications in water reuse and desalination, it is necessary to discover new and better design of LbL with appropriate electrolytes and cross-linkers.

Redesigning FO membrane structure by coating another relatively thin layer below the porous substrate called double-skinned membrane is another route to improve membrane performance for promotion from laboratory-scale uses to real applications. Double-skinned membranes have a typical sandwiched structure with two active layers on both sides of the substrate to improve the performance and antifouling properties. The primary active layer provides rejection ability, while the other one acts as an antifouling barrier that prevents the entry of foulants to the substrate. Meanwhile, the properties of the porous substrate layer sandwiched between two selective active layers are maintained. The possibility of redesigning and reengineering of membrane structure into double-skinned membrane or sandwich-like membrane would resolve the dilemma of asymmetric membranes to either suffer severely from dilutive ICP (in AL-FS mode) or have greater fouling tendencies (in AL-DS mode). The study deduced that compared to the single-skinned counterpart, double skinned membranes gradually showed better advantages due to greater controllability [60]. Therefore, the double-skinned strategy potentially reduced the adverse influence of ICP within the membrane porous substrate layer.

## 5. Recent Progress and Performance Evaluation of TFC-FO with Modified Substrate

For simplicity, this review proceeds according to the aforementioned techniques. A few significant studies in the literature based on modification techniques to obtain desirable TFC-FO membrane are reviewed. The effects of these modifications on the membrane properties and performance particularly on wastewater and desalination applications are briefly discussed in the following sub-sections. Comparison of the research findings obtained through the two modes of membrane orientations (according to their application and modification techniques) are also included in the summarizing tables of the next section.

### 5.1. Electrospun Nanofibers

**Figure 4:**
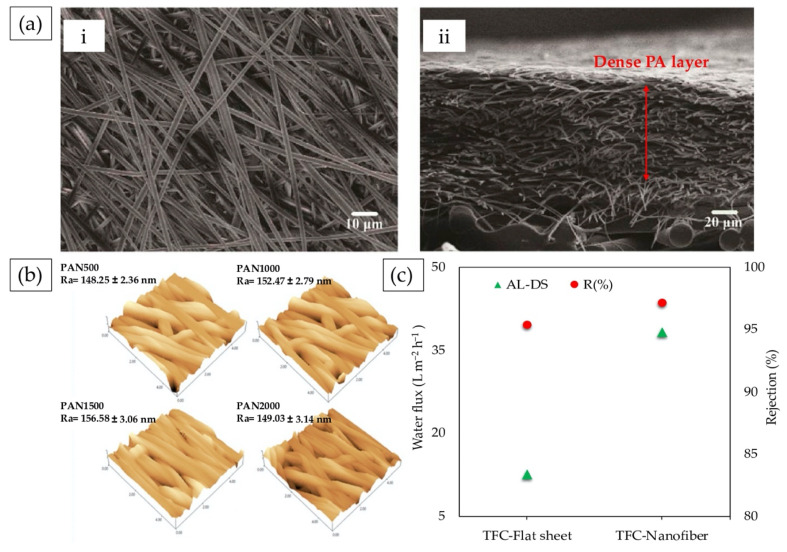
(**a**) SEM images of the polysulfone (PSf) nanofiber substrate (i) top surface and (ii) cross-sectional dense polyamide (PA) layer [43]. (**b**) Surface topologies of polyacrylonitrile (PAN) nanofiber substrates prepared at different rotating speeds of 500, 1000, 1500, and 2000 rpm [150]. (**c**) FO performance of the prepared TFC flat sheet and nanofiber membranes [51].

As confirmed experimentally, the best membrane performance in the FO process could be achieved when the membrane has a minimal *S* value and good anti-fouling properties [59]. Electrospun membranes are a promising choice for developing high-flux FO membranes as their interconnected pore structure permits a shorter path for diffusion of molecules, allowing a higher flux to be obtained. Earlier, the studies by McCutcheon et al. demonstrated that nanofiber-supported TFC-FO membranes exhibited smaller *S* and excellent water flux, owing to the porous and less tortuous nanofibrous substrate layer [115,149]. In a recent work, Zhang et al. [43] proposed nanofibrous TFC-FO membrane to treat wastewater containing six antibiotics, with the antibiotic rejection mechanism being investigated systematically. Using PSf as the polymer matrix, the nanofibers were obtained by electrospinning, hot-pressing (compacted), and the interfacial polymerization process. As a result, the PSf electrospun nanofiber substrate displayed uniform fibers with smooth surfaces (Figure 4a(i)). Compared with the uncompacted nanofiber substrate layer, the nanofibers produced a good hydrophilicity and mechanical properties that were of benefit to the preparation of a dense active layer on the membrane surface, as exemplified in Figure 4a(ii). Consequently, the membrane exhibited excellent water flux of 49 L m^−2^h^−1^ and antibiotics compound rejection of >98% using 1M NaCl solution as draw solution. It was deduced that the antibiotic rejection for neutral and macromolecular-charged antibiotics was mainly dominated by steric hindrance. On the other hand, small molecular-charged antibiotics were affected by electrostatic attraction.

Han et al. [150] broke the bottleneck of ICP in FO separation by designing a high-performance nanofiber membrane–PAN nanofiber substrate with a slit-shape pore structure. During the electrospinning process, the authors randomly orientated and uniaxially aligned substrates at rotating speeds of 500, 1000, 1500, and 2000 rpm. TFC membranes were then prepared by forming a thin PA active layer on the as-prepared PAN nanofiber substrates. TFC nanofiber with highest degree of nanofiber alignment derived from 1500 rpm speed was entitled for well-aligned nanofiber substrate (larger pore size and better pore interconnectivity). Surface topology, shown in Figure 4b, verified that PAN1500 nanofiber substrates displayed a relative high roughness of 156.58 nm, which contributed to the better hydrophilicity. Regarding the lower permeation resistance of aligned nanofiber substrates with slit-shaped pores, higher porosity, and hydrophilicity, as well as the thinner PA active layer, the membrane exhibited effectiveness in ICP. Taking advantage of the resultant TFC-FO membrane, the structure achieved the best water fluxes of 50.7 (AL-FS) and 62.9 L m^−2^h^−1^ (AL-DS), accompanied with the ultralow reverse salt flux using deionized water feed solution and 1 M NaCl aqueous draw solution. Tensile strength results concluded that at 1500 rpm, nanofiber substrate achieved 236% improvement compared with that of 500 rpm because the aligned membrane did not rupture easily during the stretching process.

To confront with porosity reduction issue, researchers have explored electrospun nanocomposites to benefit from the unique properties of other material, while retaining the advantages of nanoscale features. Novel TFC-FO nanofibers were fabricated by electrospinning a mixture of PSf and PAN nanofibers and comparing them with the PSf/PAN substrate fabricated by phase inversion [51]. Nanofiber TFC demonstrated significant improvement in hydrophilicity and water permeability; meanwhile, the reverse salt flux was reduced due to the formation of uniform PA layer with better adhesion than the conventional substrate. Additionally, high porosity with higher specific surface area and open pore structure of nanofibers induced the low *S* of 340 µm compared to conventional TFC membrane; 1230 µm represented the reduction ICP during the FO process. The evaluation performance showed the same osmotic pressure; using 1 M NaCl as draw solution, the electrospun nanofiber membrane exhibited higher water flux (38.3 L m^−2^h^−1^) and retained a relatively high salt rejection in comparison with that of the TFC membrane (12.6 L m^−2^h^−1^). Their comparative performances in terms of water fluxes and rejection under AL-DS orientation is presented in Figure 4c. To achieve the best possible performance, Park et al. [151] fabricated TFC-FO membranes using electrospun nanofiber supports using PVDF. The hydrophobic PVDF nanofiber substrates were subsequently hydrophilized via polyvinyl alcohol (PVA) dip coating before PA selective layer was deposited on the nanofiber. Interconnected pore structure accompanied with high porosity and hydrophilicity of the resultant membrane collectively alleviated the effect of ICP, as confirmed by a low *S* value of 154 µm, while increasing the mechanical properties. The TFC-FO membrane hence exhibited high water flux of 24.8 L m^−2^h^−1^ in AL-FS mode, and 32.6 L m^−2^h^−1^ in AL-DS mode with low reverse salt flux using 1 M NaCl as draw and deionized water (DI) water as feed solution.

### 5.2. Blending

#### 5.2.1. Sulfonated Polymer Blending

Numerous studies have been performed by using sulfonated polymer or addition of sulfonated polymer into polymer substrates for desalination purposes [85,152,153,154]. One of the earliest studies by Widjojo et al. [48] showed that blending sulfonated polymer is a feasible protocol that showed encouraging outcomes. They successfully fabricated membrane with different morphologies and degrees of hydrophilicity by varying the amount of sulfonated material in the substrate. Interestingly, the best performance was acquired from a more sponge-like hydrophilic structure. Sulfonate groups can be introduced into polymer by simple blending [39]. It is worth noting that both materials and sulfonation degree need to be considered since they provide beneficial influence on morphology and water holding capacity. It has been reported that beyond 70% sulfonation degree (strong hydrophilicity), sulfonated-based substrate exhibits poor flexibility and an over-swollen nature due to excessive water adsorbed by sulfonic groups [85]. In addition, the membranes could suffer from inferior mechanical strength, which limits their potential application.

The integration of sulfonated polysulfone (SPSf) with commercial polyester nonwoven fabrics were first considered by Ren and coworkers [85] to fabricate high performance TFC-FO membranes for desalination. Different degree of SPSf, i.e., 9% and 16%, were introduced into PSf via blending-phase inversion. It was found that the sulfonated blended membrane increased the surface hydrophilicity because the sulfonic acid replaced the hydrogen atoms in an electrophilic interaction, hence consequently promoting water transport during osmotic processes. Addition of 16% SPSf in dope solution exhibited the best water flux of about 69.4 L m^−2^h^−1^ using 1 M NaCl against deionized water in PRO mode. This can be attributed to the improvement in the membrane structure and properties such as a thinner and porous membrane supporting structure with less tortuous finger-like morphology that accelerates the salt diffusion to alleviate the dilutive and concentrative ICP. Besides this, thin SPSf of the substrate layer is useful to maintain the mass transfer resistance whilst thick PET imparts better mechanical durability.

Similar observations have been reported by Sahebi et al. [155], who anchored hydrophilic sulfonated polyethersulfone (SPES) into the PES membrane substrate to enhance water flux in the FO membrane. Different sulfonation degree of SPES: 0 SPES, 25 wt % SPES, and 50 wt % SPES were blended with PES. Blending a high degree of SPES caused a delay in demixing, which resulted in morphology changes. While neat membrane clearly showed the presence of finger-like pore structures, the introduction of 50 wt % SPES suppressed the formation of these finger-like structures and formed a sponge-like support structure. The morphology not only provides better resistance to membrane compaction, but also formed a smooth PA layer. Such changes were accompanied with increased hydrophilicity, leading to improved permeate fluxes and lower *S* parameter of 245 μm, with 77% reduction. These findings revealed that finger-like substrate structures are not the major contributor to higher water flux; instead, increasing hydrophilic characteristics of substrates is a much stronger factor in enhancing the water flux of FO membrane.

Zheng et al. [153] used inexpensive poly(vinyl chloride) (PVC) polymer blended with sulfonated polysulfone (SPSf) as a hydrophilic modifier. It was reported that the most permeable membrane with desired hydrophilicity, morphology structure, and *S* parameter was obtained when 2.5% SPSf was added in the polymeric solution. In both AL-FS and AL-DS configurations, water flux increased more than 80% while the specific salt flux exhibited a low value of 0.10 gL^−1^ in AL-FS and 0.09 gL^−1^ in AL-DS. This can be ascribed to the looser and more porous substrate layer upon the addition of hydrophilic PVC, thereby providing a short path from the bulk draw solution to the active surface of the membrane, and hence lowering the ICP. Nonetheless, further increase in SPSf concentrations up to 10% reduced the FO efficiency due to the fact that high sulfonation degree is susceptible to swelling. The lower blend ratio of 2.5% SPSf is hence the ideal form on the basis of the intrinsic properties and superior FO performance in terms of salt flux and water flux results coupled with manufacturing costs of membrane fabrication.

Another important factor affecting the structure and properties of blend membranes is the compatibility among the polymers. Zhang et al. [64] modified PVDF substrate by blending with hydrophilic perfluorosulfonic acid (PFSA), with concentration ranging from 1 to 5 wt %. The compatibility of PFSA and PVDF led to formation of a membrane with improved surface porosity and wettability. In addition, PFSA induced a more perfect formation of the PA top layer with enhanced selectivity as salt rejection increased sharply by 83%. Ascending content of PFSA up to 3 wt % contributed a desirable porous finger-like morphology and high hydrophilicity that promoted water transport and solute diffusion across the substrate layer. Consequently, the best performance in terms of water and reverse salt flux in FO and PRO modes were observed for the 3 wt % PFSA membrane using deionized water and 1 M NaCl as the feed solution and draw solution, respectively. Moreover, the *S* drop was more significant, from 1606.51 μm for membrane without the PFSA in the substrate to 334.62 μm, which can be reasonably associated with the positive impacts brought by PFSA in PVDF substrate, laying the foundation of the enhanced FO performance of the resultant TFC-FO membrane.

**Figure 5:**
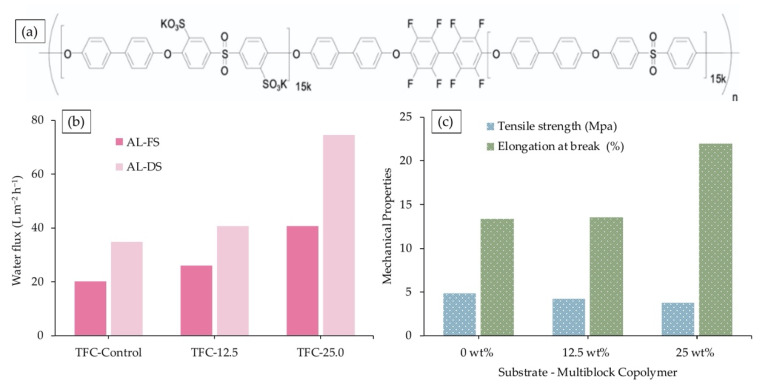
(**a**) Chemical structure of disulfonated poly(arylene ether sulfone) multiblock copolymer. (**b**) Water flux of membranes at different multiblock copolymer contents using water as feed and NaCl as draw solution. (**c**) Mechanical properties of substrate with 0 wt %, 12.5 wt %, and 25 wt % multiblock copolymer [120]

In addressing the general issue of sulfonated polymer blending, polymer elution, and low mechanical property due to swelling, Zhang et al. dispersed disulfonated poly(arylene ether sulfone) multiblock copolymer at different content levels (12.5 wt % and 25.0 wt %) along with PSf polymer in casting solution prior to membrane casting followed by PA layer formation for seawater desalination [120]. As shown in Figure 5a, the copolymer consisted of hydrophilic (sulfonated)–hydrophobic (unsulfonated) segments, which are arranged alternately in the structure to tailor membrane compatibility, hence increasing the hydrophilicity and porosity of the substrate. Consequently, water fluxes of TFC-25.0 showed an improvement of nearly 100% (Figure 5b), with reasonably acceptable reverse salt fluxes, using 2.0 M NaCl as the draw solution. The contribution of the high-water flux was dominated by remarkable reduction of the ICP effect in the porous substrate, where the *S* value decreased from 1011 µm to 397 µm. Figure 5c presents results from a mechanical properties viewpoint, where hydrophilicity preserved the mechanical strength of substrate, as only a slight decrease was observed in tensile strength for substrate containing high (25 wt %) multiblock copolymers. Moreover, the modifier endowed the membrane with great flexibility (highest elongation at break) that suits the need of industrial handling and large-scale applications.

#### 5.2.2. Pore Former Blending

Characteristics of final membranes fabricated via phase inversion are influenced by a variety factors including the presence of pore-forming additives that are well known in creating a porous membrane structure even with small addition [66]. Macromolecules and hydrophilic polymers, i.e., PVP, PVC, PEG, and inorganic salts, are commonly used pore forming additives. They help to promote pore formation and structure in the polymeric membranes, enhancing their permeation properties through the changed viscosity of the dope solution and the exchange rate between the solvent and non-solvent during the phase inversion process [94]. Wu et al. [125] prepared substrate of TFC-FO membranes using PSf (Mn 22000) and PEG (Mw 400) as additives. The increase of the PEG-400 content from 0 to 6 wt % altered the rate of phase separation, which ultimately influenced the structural morphology, wherein more large pores were observed. However, the further increment of PEG impaired the FO performance due to rheological hindrance to non-solvent intrusion during phase inversion and high viscosity of casting solution that suppressed large pores in substrate. Apparently, the presence of optimal PEG-400, 6 wt %, considerably reduced the ICP and produced better membrane performance by means of enhancing membrane hydrophilicity, porosity, and pore area percentage within substrate layer. The membrane exhibited relatively higher water flux and salt rejection of 47.4 L m^−2^h^−1^ and 95.60 % using 2.0 M NaCl draw solution.

Polyvinyl butyral (PVB) with concentrations ranging from 0% to 4% was used as an additive of cellulose acetate butyrate (CAB) substrate [156]. As the concentration of PVB increased from 0 to 2 wt %, pores were transformed from a sponge-like to a finger-like macrovoids structure. Substrate with high porosity and high pore connectivity as well as finger-like pore structure was formed to facilitate mass transport and suppress the ICP. Consequently, the water flux was enhanced by 130% compared to an unmodified counterpart, accompanied with small specific salt flux of 0.35 gL^−1^. The membrane showed the lowest fouling tendency with the highest water flux recovery of > 98% when tested with feed solution containing bovine serum albumin (BSA) as foulant. Besides this, the effect of inorganic salts such as lithium chloride (LiCl) as pore former has also been investigated for TFC-FO membrane. It has been evidenced that LiCl minimized the influence of ICP, thus rendering high water flux, regardless of the low tortuosity and high hydrophilicity [65,157,158]. Similarly, Li and colleagues [158] successfully fabricated hollow fiber substrate with minimal tortuosity and dimension. As evident from their morphological studies, LiCl present in the support layer decreased the mean pore size and enhanced the porosity in comparison to support layers without LiCl. Optimum use of LiCl at a concentration of 3.5 wt % contributed to water flux of 31.8 L m^−2^h^−1^, which was 25% higher than that of TFC membrane when tested with 1 M NaCl as draw solution and DI water as feed solution in AL-FS orientation. Similarly, Darwish et al. [65] incorporated LiCl into three porous substrates, namely, PSf, PES, and PPU in order to accelerate the pore formation and improve pores interconnectivity for enhanced FO desalination performance. Water flux of the TFC flat sheet membrane increased by 20.85, 21.57, and 21.95% upon 3 wt % LiCl addition of PSf, PES, and PPSU, respectively, compared to TFC membranes without the additives. Nevertheless, the high reverse salt flux observed in this study also implied that further fine-tuning is needed to increase the water flux without compromising the reverse salt flux.

**Figure 6:**
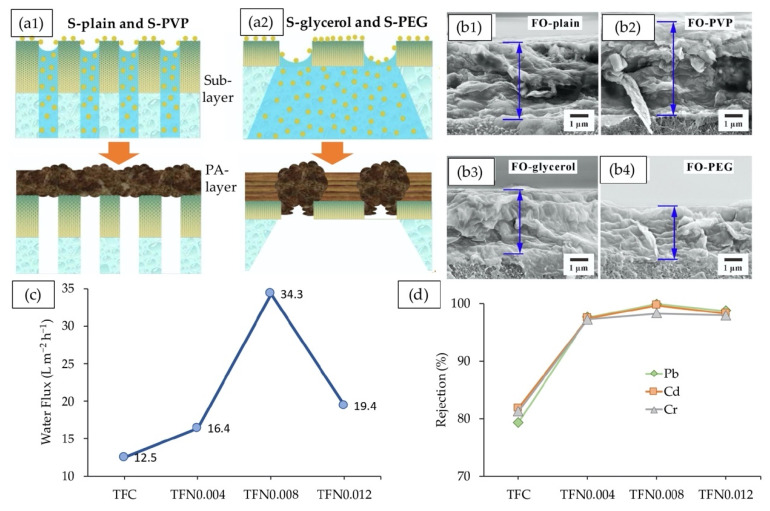
The effect of different structures of substrates on PA layer formation (**a1**,**a2**) and formation foulants layer on the membrane surfaces (**b1**–**b4**) [79]. (**c**) Water flux behavior and (**d**) rejection of heavy metals from synthetic wastewater using different membranes [159].

In a recent study, the effects of substrate structural properties on the in situ PA layer formation and anti-fouling capacity of the TFC-FO membrane were investigated by adopting various additives, PVP, and PEG into the SPES matrix [79]. As illustrated in Figure 6a,b, different structures of substrates produced varying PA layers and ultimately foulant growth. Correspondingly, the sponge-like PEG substrates enabled the formation of a highly cross-linked, thinner, and smooth PA layer while a thick, rougher, and less dense PA layer was inclined to form on macroporous PVP substrates. Benefiting from the surface pattern with fewer carboxylic groups, the TFC-FO membranes with PEG exhibited low foulants (organic foulants and multivalent metal ions) coverage and hence offered the highest average water flux (35.08 L m^−2^h^−1^) and lowest drop of water flux (0.525) throughout multicycle concentrating performance in domestic wastewater. Higher rejection of TOC (97.48%) and PO_4_^3−^-P (98.53%) were observed for FO-PEG, however at a poor NH_4_^+^-N rejection, much like other as-fabricated membranes, hence being in need of further exploration. The selectivity and performance of TFN-FO membranes was enhanced by modifying both support and active layer with additives PEG-400 and graphene oxide (GO), respectively, for wastewater treatment [159]. Membranes were cast using dope solution of PSf and PEG-400 (weight ratios of 0, 2, 4, and 8%). TFN-FO membranes were subsequently prepared by interracially polymerizing a GO-modified PA layer on each substrate to enhance hydrophilicity. Conversion of bottom surface from finger-like pores to large macrovoids was observed for the TFN membrane containing optimal amount of 4% PEG and 0.008% GO. The change, in turn, improved the hydrophilicity and porosity, and thereby *S* value became smaller (119 μm) while water flux remarkably increased by 174%, which is comparable to that of TFC-FO membrane with no PEG and GO (Figure 6c). As depicted in Figure 6d, the TFN membrane was also found to effectively reject Pb, Cd, and Cr in the synthetic wastewater with rejections of 99.9, 99.7, and 98.3%, respectively.

Similarly, attempts to improve wastewater treatment using TFC-FO membranes have been thwarted by ICP, antifouling, and low flux. Several other strategies are ongoing to improve the performances of the TFC-FO membrane for power regeneration and wastewater applications by employing different and innovative materials. High-performance TFC-FO membranes have been prepared on the basis of PSf-sulfonated polyether-ether ketone (SPEEK) blend substrates through different approaches, including single-layer casting and co-casting for concentrating high-salinity wastewaters [160]. The SPEEK was able to uphold high water capacity that was linked to the behavior of high proton conductivity, resulting in stable hydrophilized membrane substrate [58]. Hydrophilicity of the PSf substrate was tuned by blending with 0.5 wt % SPEEK. In contrast to PSf and SPSf substrates prepared through single layer casting, the TFC FO membrane prepared via co-casting (SPSf) showed a bottom surface with an open-pore structure, which featured relatively better hydrophilicity and higher porosity. The resultant membrane yielded excellent intrinsic separation properties (pure water permeability coefficient: 2.16 L m^−2^h^−1^; solute permeability coefficient: 0.16 L m^−2^h^−1^; *S* value: 191 μm), thereby offering a low ICP effect. Further application of the hydrophilic TFC membranes in the treatment of high salinity wastewaters (10 wt %) demonstrated the highest water recovery rate of 53.2%, which was in line with the FO water flux (28.3 L m^−2^h^−1^) observed, in comparison to the neat TFC membrane and TFC through the single-layer casting. The overall results indicate hydrophilicity effectively contributes to improvement of water permeability.

Cui et al. prepared TFC membranes consisting of a PA layer and a porous substrate cast from three different materials: Matrimid, polyethersulfone (PESU), and sulfonated polyphenylene sulfone (sPPSU) using similar solvent, n-methyl-2-pyrrolidone (NMP) for the removal of organic micropullants from wastewater [161]. Dope formulations are PESU/PEG 400/NMP/H_2_O with a weight ratio of 20.4/37/37.7/4.2, Matrimid /PEG 400/NMP with a weight ratio of 18/16/66, and sPPSU/PEG 400/NMP with a weight ratio of 15/27/58. The performances of all three TFC membranes to all the organic micro-pollutants under the FO processes are higher than in RO mode using 1000 ppm aromatic aqueous solution and 1 M NaCl as feed. The sequence under the FO mode is sPPSU > PESU > Matrimid when comparing the flux performances of the three TFC membranes. As the best-performing substrate layer, the sPPSU TFC membrane had the highest water flux (22.0 L m^−2^h^−1^), due to its hydrophilicity and smaller thickness compared to the others. Further thickness increment of substrates PESU (≈70 µm) and Matrimid (≈65 µm) lowered their water flux by 20.6 and 14.4 L m^−2^h^−1^, respectively. In addition, the rejection of pollutants was statistically comparable and all consistently higher than 72%. The values followed the order of aniline > nitrobenzene > phenol.

#### 5.2.3. Carbon-Based Nanofiller Blending

By virtue of their antimicrobial, high efficiency, potential energy savings, and ease of integration and scale-up, carbon-based nanomaterials, especially activated carbon, graphene oxides (GO), and carbon nanotubes (CNTs) in various derivatives, have seen an upward trend in a wide range of separation applications [4,111,162,163,164]. Many researchers have joined efforts to unveil CNTs and GO potential applications by compositing in a substrate or active layer of membrane to produce TFC membranes with the ability to reduce ICP and fouling tendency along with enhanced hydrophilicity, permeability, and solute rejection [121,122,139,165,166]. Earlier, Choi et al. [82] studied the efficiency of polyamide TFC supported on functionalized multiwalled CNT (MWCNT)-blended PES substrate for integrated seawater desalination and wastewater recovery through the FO process. The substrate of the TFC membrane possessed greater porosity and hydrophilicity derived from inherent characteristics of MWCNT. This was confirmed by high water flux, corresponding to 72% improvement of water flux over the unmodified TFC membrane. Meanwhile, carboxyl and carbonyl groups of MWCNT on the support surface endowed negative charges, which in turn improved the electrostatic repulsion between foulants and surface of substrate layer. The smoother surface the membrane, which was induced by affinity of MPD solution towards hydrophilic substrate, improved fouling reversibility and subdued the alginate-fouled membrane rates. The accumulation of alginate foulants on the TFN-CNT membrane was less and could be easily removed after physical cleaning compared to TFC and commercial TFC membranes. The recovered normalized flux was observed to be 6% higher than that of TFC membrane after physical cleaning. As underpinned in other studies, favorable membrane changes by CNTs have proven to improve the level of separation performance with great membrane stability [4,121,167].

Since the oxidization process of MWCNT was attributed to severe destruction that restricts membrane performance, the route of MWCNT modification has been switched to silane functionalization of MWCNTs in order to incorporate oxygenic functional groups in MWCNT. Then, for the first time, Zhang et al. [168] blended the hybrid nanofiller, SiO_2_@MWCNT with PVDF polymer, with the expectation that the nanofiller will dispersed well into the matrix. With the nanofiller loading of 0.75 wt %, the resultant membrane appeared to offer a uniform and rougher PA layer as a result of better dispersion of SiO_2_@MWCNT in PVDF, in turn promoting high hydrophilicity and porosity of the modified substrate to allow high loading of MPD on substrate. Besides boosting water permeation events into the substrate, SiO_2_@MWCNT also facilitated water molecule migration across the membrane substrate, hence controlling the ICP phenomenon. When tested using DI water as feed and 2 M NaCl as draw solution, the membrane demonstrated high salt rejection of 96.3% and optimal water flux of 29.5 L m^−2^h^−1^, which was greater than the flux of TFC-FO membrane (for AL-DS). Meanwhile, the excess SiO_2_@ MWCNT loading (1 wt %) led to unfavorable membrane properties and performance due to agglomeration in the substrate.

Meanwhile, Aziz et al. [169] blended protonated graphene-like carbon nitride nanosheets, which were obtained from acid treatment of carbon nitride with PSf substrates. A more hydrophilic and wettable surface was obtained from the substrate modification. Interaction of hydrogen bonding of water molecules with the nitrogen atoms of carbon nitride promoted high wettability of the nanocomposite substrate. The resultant TFN produced better water flux of 11.0 L m^−2^h^−1^ and lower reverse solute flux of 0.3 g m^−2^h^−1^ in AL-DS mode compared to TFC membrane. The reduction of *S* from 27.4 mm to 2.96 mm further confirmed the suppression of ICP.

#### 5.2.4. Metal Oxide-Based Nanofiller Blending

In addition to the aforementioned carbon-based nanomaterials, a simultaneous improvement of membrane water permeability and reduction in structural parameter can be realized by the metal oxide based-nanocomposite membrane approach. Nanostructured metal oxides such as TiO_2_, MnO_2_, Fe_3_O_4_, ZnO, or Al_2_O_3_ have been considered as the focal point of many researchers in order to treat specific contaminated waters, owing to large surface area, high activity, and high adsorption capacity [9,170,171]. Compared to purely polymeric membranes, the metal oxides incorporated in membrane developed excellent properties, including membrane hydrophilicity, photocatalytic activity, antifouling properties, and permselectivity, which eventually heightened the membrane performance. Comprehensive past research has demonstrated that the incorporation of TiO_2_ as nanofillers into the TFC substrate tuned the support layer morphology and physiochemical properties to ameliorate the water flux because of exclusive features, i.e., high hydrophilicity, chemical stability, good anti-fouling, and commercial availability [137,172,173]. The effects of TiO_2_, GO, and mixture of both as nanofillers on the properties of PSf substrate was investigated by Sirinupong and coworkers [137] for FO desalination application. It was reported that incorporation of both TiO_2_ and GO nanofillers into PSf substrate rendered high surface hydrophilicity, porosity, thermal stability, and mechanical strength of the membrane. As a result, pure water flux relatively increased along with the addition of nanofillers: TFN–TiO_2_/GO (297.7 L m^−2^h^−1^) > TFN–GO (201.6 L m^−2^h^−1^) > TFN–TiO_2_ (140.5 L m^−2^h^−1^) > TFN_control_ (110.2 L^−2^m^−1^h^−1^). Meanwhile, water flux of the optimal nanofiller-modified membrane was 25–50% more than that of TFC membrane using DI water as feed side and 2 M NaCl as a draw solution. Salt rejection of membrane slightly decreased from 96.0% (TFC_control_) to 94.4, 91.1, and 90.1% for TFN–TiO_2_, TFN–TiO_2_/GO, and TFN–GO, respectively, with no significant increase in reverse draw solute flux.

Darabi et al. [134] controlled ICP by adding hydrophilic Fe_3_O_4_ as a nanofiller in the PES dope solutions. The pronounced hydrophilicity and overall porosity due to diffusion of nanofiller toward the coagulation bath during phase inversion were the factors behind the results improvement as they were directly connected to the *S* parameter and suppressed the ICP. The presence of optimal nanofiller, 0.2 wt % Fe_3_O_4_, yielded a high-water flux with low specific reverse salt flux, indicating and a more favorable *S* value of 0.42 mm in both AL-FS and AL-DS orientations using 10 mM NaCl and Caspian seawater as feed solution, with 0.5 or 2 M NaCl used as draw solution. It was found that a greater amount of Fe_3_O_4_ (> 0.2 wt %) resulted in flux decline (low FO selectivity), likely due to pore blocking and nanoparticle agglomeration. In a similar vein, Darabi et al. [174] fabricated TFN-FO via interfacial polymerization by introducing nanocomposite Fe_3_O_4_-photocatalytic nanoparticles, ZnO to diminish ICP, and fouling of TFC-FO membranes for pharmaceutical wastewater treatment. The nanocomposite was blended with PES substrate (0.1, 0.2, and 0.3 wt %) and active layer (0.02 wt %). The membrane with a low concentration (i.e., 0.2 wt %) of Fe_3_O_4_-ZnO offered the highest salt retention (96.5%) with water flux 78% higher than the TFC membrane and a lower *S* parameter of 400 µm against 10 mM NaCl feed. The superior ZnO anti-fouling behavior prevented remarkable initial flux decrement after 8 h fouling test. An increase in hydrophilicity upon UV irradiation, porosity, and water passages (facilitating water transport) of substrate were found to be the significant factors to such performance, indicating that nanocomposite membranes suffer a less intensive ICP effect.

The incorporation of porous nanofillers such as metal organic frameworks (MOFs) have also been attempted to tailor TFC membrane performances. Facilitated by the metal ions and organic bridging ligands, MOFs offer diverse structures and functions over other porous materials such as zeolites and carbon-based materials. The adjustable chemical functionality, mild preparation conditions, and extremely porous structure make MOFs viable candidates in terms of achieving desirable finger-like or needle-like structures for higher mass transfer of water molecules [133]. In the investigation performed by Arjmandi et al. [175], highly porous MOF-5 was used to tailor the physical structure of TFC substrate. MOF-5 nanofillers constitute a synthesis of various conditions rendering a porous matrix membrane blended with different polymers, i.e., PES and polyetherimide (PEI). The extent of crosslinking between PA layer and substrate layer increased, owing to a larger pore size of resultant substrates. Judging from the transport parameters and salt rejection of PEI-based TFC membrane, MOF-5 synthesized at 25 °C cooling temperature was effective in terms of altering the performance of the TFC-FO membranes. The rejection of salt was up to 98.42%, whilst the *S* parameter showed 38% reduction because of the highest degree of crosslinking (97%) and largest pore size of the support layer. In addition to this, the search for nanofillers that have been reported in literature may be broadened to include other kinds of MOF, mesoporous, or microporous materials in order to improve permeability and salt retention of FO membranes, despite some hurdles yet to be overcome.

### 5.3. Template-Assisted Fabrication

Li et al. [176] efficiently designed a silica template strategy to address this ICP issue, which is prepared by incorporating silica nanoparticles into the PES substrate. Researchers have incorporated silica into polymer matrix to obtain good features of silica, such as a non-toxic, hydrophilic nature; uniform pores; highly porosity; and good thermal stability, which can improve the water permeability of membranes [177,178,179]. However, to resolve common filling issues of segregation as well as porosity blocking, the as-encapsulated silica has been completely removed by hydrofluoric acid (HF) etching, leaving substrate with highly porous and interconnected-pore structure. Such porous structure favors the salt back diffusion in the substrate layer, further improving the performance of the membrane. HF treatment also contributes to a more hydrophilic top polyamide layer, further improving the performance of the membrane. Highly permeable membrane and threefold higher water flux was recorded for TFC with 5 wt % silica after HF treatment compared to neat TFC membrane. This template-assisted fabrication, however, requires a more environmentally friendly solvent rather than HF in terms of its use in the etching process.

Zhao et al. [143] initiated the templating approach using ZnO by preloading the nanoparticles in the PVDF and subsequently etching them by 1 M HCl. The improved porosity and optimized pore microstructure–TFC membrane showed 50% higher water flux with comparable NaCl rejection compared to the unmodified membrane. Changes of *S* value from 2223 µm (neat TFC) to 413 µm (ZnO-TFC) verified that etching ZnO template exerts influences on thickness, porosity, and tortuosity of the substrate and consequently inhibits the ICP. Likewise, Rastgar and co-workers [142] embedded the ZnO into the PES matrix to elevate the membrane separation performance. ZnO is known as a non-toxic material that offers controllable morphology; therefore, they utilized two different ZnO nanostructures—nanoparticles and nanorods—to manipulate the pore structure in PES. Nanorods with a larger size were more beneficial in enhancing permeation flux and reducing ICP effect (lowest *S* value), owing to higher hydrophilicity and larger pore size that were highly interconnected for easy optimization of water flow channel. After acid washing, the membrane (2 wt % of ZnO nanorods) had a water flux of 31.78 L m^−2^h^−1^, which was 34% more than the value achieved in the membrane modified by 2 wt % of ZnO nanoparticles.

**Figure 7:**
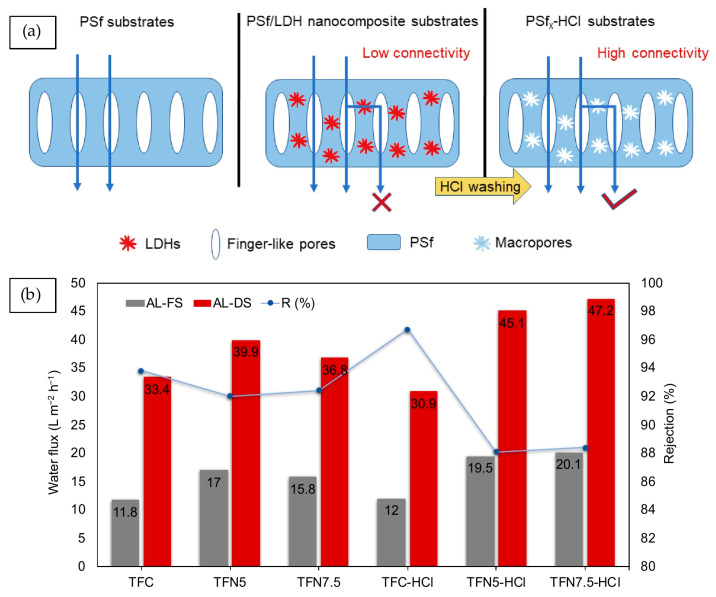
(**a**) Conceptual images of templating approach by preloading layered-double hydroxide (LDH) inside PSf. (**b**) Membrane with 7.5 wt % LDH etched with HCl exhibited the best of flux and NaCl rejection [180].

Recently, another example of the template-assisted method was reported by Lu et al. [180], where three-dimensional (3D), layered-double hydroxide (LDH) was used as a novel macropore template to form PSf substrate with a high porosity and connectivity. LDHs, on account of their high hydrophilicity, anion exchange capacity, surface areas, and flexible interlayer region have drawn tremendous interest in improving the efficiency of membranes. The addition of LDH would enhance the efficiency of membranes with respect to antifouling and permeating flow. A well-developed layered structure of 3D LDH was achieved. The presence of LDH provided serious aggregation in the substrate, and hence HCl was used to etch LDH leaving only PSf substrate with HCl. As illustrated in Figure 7a, the dissolving of LDH left behind macropores with spheroidal sand rose structures. The templated macropores enhanced the overall porosity and pore connectivity, hence reducing ICP in the FO process. Substrate embedded with 7.5 wt % LDH exhibited the highest water flux of 20.1 and 47.2 L m^−2^h^−1^ in AL-FS and AL-DS modes, respectively. On the basis of the results (Figure 7b), the modified substrate also increased salt rejection and decreased the *S* value, implying that the microstructural etching of substrate can effectively improve of the separation performance of the FO desalination membranes.

### 5.4. Surface Coating

#### 5.4.1. Interlayer Coating

Another possible approach to enhance membrane efficiency for FO applications is by decorating hydrophilic interlayer (sandwiched between a PA film on the top and a substrate layer on the bottom) as connection area between the substrate and the dense PA layer of TFC-FO membranes. This configuration optimizes substrate layer to reduce the effect of ICP and promotes formation of a highly crosslinked PA layer [91]. Choi et al. [147] successfully fabricated a PA selective dense layer on PSf substrate coated with a PDA/GO interlayer. Water contact angle measurement demonstrated that PDA and GO originated more hydrophilic functional groups and induced greater hydrophilicity of PSf support layer surface, thereby resulting in water permeability enhancement. The prepared TFC membrane exhibited excessive development in water flux (almost 57.6%) without a remarkable reduction in the reverse solute diffusion under an optimum condition of 0.5 gL^−1^ of GO concentration and 1 h of PDA coating time. For extended PDA coating time more than 1 h, effect of hydrophilicity membrane permeability diminished because of the pore blocking by GO as well as PDA. Overall, the interlayer on substrate proved to enhance membrane productivity (imparting high permeability for substrate and TFC membrane, hence increased flux without severe compromise of the reverse salt flux).

Kwon et al. [181] evidenced the better performance and durability of TFC-FO membrane based on polyethylene (PE) substrate modified with PDA via dip coating. A high permselective PA layer was formed on the modified PE using a toluene-based interfacial polymerization technique. The well-interconnected pores coupled with high porosity of PE support were found to be advantageous for PDA coating as the hydrophilic groups of PDA can be evenly distributed within PE support without deteriorating the structures. It was found that the *S* value was lowered to 168 μm, leading to far better FO flux of 40.7 L m^−2^h^−1^ and low specific salt flux of 0.48 gL^−1^ (in AL-FS), which surpassed HTI-CTA and HTI-CTA membranes by about 77% and 69%, respectively. Indeed, surface coating is important to uniformly hydrophilize the substrate without deteriorating the performance-affecting *S* parameter. Together with improved efficiency and functionality, the surface coated-TFC membrane inferred better mechanical and chemical properties to sustain backwash and cleaning, allowing commercialization and wide applicability in other harsh industrial environments.

**Figure 8:**
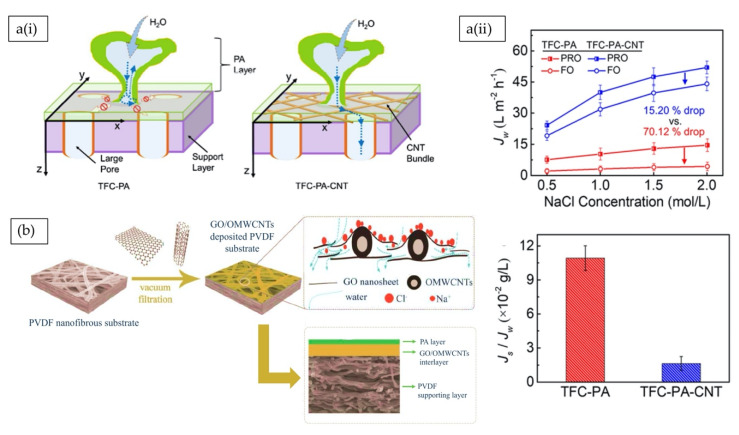
(**a**) Illustration of carbon nanotube (CNT) interlayer developing a channel for water transport on the PES substrate and the comparison of water flux [182]. (**b**) Fabrication route of graphene oxide (GO)/multiwalled CNT (MWCNT) interlayer on polyvinylidene fluoride (PVDF) nanofibrous substrates [184].

Zhou and colleagues [182] fine-tuned the surface properties of PES substrate by spray coating an ultrathin CNT interlayer between the porous PES and dense PA layer for desalination. Designing such an interlayer bestowed a smoother surface and higher surface hydrophilicity to the substrate, arising from continuous and porous CNT network structure covering the underlying PES substrate. As portrayed in the schematic illustration (Figure 8a(i)) the presence of the CNT interlayer allowed for the formation of a highly permeable and selective PA layer, as CNT bundles (interlayer) served as an alternative channel for water transport to the porous part of PES substrate. Comparatively high water fluxes were recorded for TFC with CNT interlayer in two different modes (Figure 8a(ii)). Additionally, the lower specific salt (Js/Jv ratio) obtained in PRO mode verified an excellent perm-selectivity of the PA layer formed on the CNT interlayer. CNT interlayer reduced the ICP, and hence allowed efficient separation. Further observation highlights that the reverse salt flux of the membrane was much lower (0.6 g m^−2^h^−1^) in comparison to the commercial TFC-FO membranes reported in the literature (5−35 g m^−2^h^−1^). Additionally, a stable water flux was recorded for 30 h operation, confirming a robust PA layer perm-selectivity on the TFC-CNT membrane. Other research work by Zhao et al. [183] developed an advanced TFC-FO membrane with a PVDF support layer porous membrane, a CNT network interlayer, and PA skin layer was fabricated by a facile filtration-supported interfacial polymerization technique. The fabricated FO membrane successfully reduced the consumption of the osmotic pressure caused by the substrate layer and CP problem in the filtration process.

Interestingly, in an attempt to improve the hydrophilicity of PVDF nanofibers, Yu et al. [184] developed GO and oxidized MWCNT composite interlayer onto a PVDF nanofiber mat. The composite was coated on the nanofiber via vacuum filtration method, as illustrated in Figure 8b. The interlayer exhibited ideal pathways for water molecules. Moreover, the thickness of the polyamide layer was decreased by 60%, in contrast to that without an interlayer. Therefore, as mass transfer resistance reduced, water flux increased significantly while preserving similar salt rejection. Specifically, the forming FO membrane showed super high water flux (305.89 L m^−2^h^−1^) and super low reverse salt flux (0.37 g m^−2^h^−1^) under the conditions of using deionized water and 0.6 M NaCl solution as feed solution and draw solution, respectively. It is worth mentioning that the recorded *S* value was only 82 μm when the membrane experienced only a small effect of ICP. The high performance of the fabricated membrane indicated a simple and practicable way to fabricate high-performance FO membranes for seawater desalination. Overall, the results on CNT-containing composite membrane demonstrated that the potentiality of these membranes for water treatment is highly relevant.

Liu et al. [96] prepared TFC-FO membrane with hydrophilic CaCO_3_-coated PES substrate. Polyacrylic acid (PAA) was first blended with PES to facilitate an electrostatic interaction between PAA carboxylic groups and Ca^2+^ to ensure continuous and uniform CaCO_3_ coating throughout the surfaces of the substrate. CaCO_3_ has high hydrophilicity due to ionic and hydrogen bonding with water molecules. Therefore, high hydrophilicity that is capable of decreasing ICP was imparted to the substrate upon coating. Consequently, the CaCO_3_-coated membranes were afforded enhanced water flow and effectively preserved the membrane selectivity and mechanical integrity. The effect of hydrophilicity on water flux was dominant up to 5% PAA, and the negative effect of PAA swelling became significant beyond the loading. The swelling issue of PAA, leading to contraction and pore blocking, thus resulted in the loss of water flux. Combination of PAA and CaCO_3_ with PES resulted in a water flux that was 3.25 times higher (AL-FS mode) than that of the PES membrane.

#### 5.4.2. Layer-by-Layer Assembly

Suwaileh et al. [45] performed a simple vacuum-assisted filtration method to generate LbL film onto PES substrate for brackish water desalination. Previously, Su et al. [185] applied a multilayer with a high number of polyelectrolytes to increase selectivity toward multivalent salts and organic molecules, a process that is very time consuming. In this modification protocol, poly(ethylenimine) was first crosslinked with hexadecafluorodecanedioic acid and deposited on PES substrate followed by PAA anionic layer in alternate sequence, performed in order to render positively charged membrane surface with high stability and minimum scaling. Accordingly, a homogenous and uniform selective layer was formed on the substrate, which significantly improved the hydrophilicity of the substrate. Surface charge LbL membrane was desirable at a high positive charge, wherein at pH 7, the value approached 6.9 mV (4.5 bilayer) as compared to the pristine membrane with a zeta potential value of approximately −11.0 mV. Optimum 3.5 bilayer contributed to a 22.48 L m^−2^h^−1^ water flux (distilled water as feed and 2M NaCl as draw) followed by 10% reduction as each successive polyelectrolyte bilayer was deposited. The circumstance was attributed to the smaller average pore size due pore clogging phenomena by polyelectrolyte multilayers, which in turn lowered the reverse solute flux by approximately 20% compared to neat membrane. Further experiments are necessary to optimize cross-linker concentration, the stoichiometric ratio between PEI and HDFA, and cross-linking time.

Kang et al. [186] assembled LbL membrane on PES substrate by utilizing two ideal nanomaterials, GO and oxidized CNTs, linked using poly(dimethyldiallylammonium chloride). Besides retaining a GO-ordered structure in LbL caused by NaCl, such a combination was a strategy to inhibit agglomeration of GO in the LbL membrane. Five bilayers of GO-CNT LbL membrane showed stronger hydrophilicity, with a water contact angle of 42.5° regardless of combination of hydrophilic functional groups from GO and CNTs. Hence, the membrane exhibited much higher water fluxes while solute flux (NaCl) dropped to about 70.2% of that of membrane assembled by GO. Inarguably, OCNTs played an important role in stabilizing the structure of the GO–CNT–LbL membrane as well as enhancing the performance.

#### 5.4.3. Double-Skinned/Sandwiched-Like Membrane

Double-skinned membranes with two selective layers on both sides of the substrate have been developed. For instance, Song et al. [187] preparing double-skinned FO membrane with PDA/CNTs (0.01, 0.05, 0.1 w/v %) coating on both the top and the bottom layer of PSf substrate. Both the top surface and bottom surface of the membrane underwent interfacial polymerization reaction. Two different orientations, top active layer facing feed solution (AL-FS) and top active layer facing draw solution (AL-DS), were used in evaluating FO performances. The double-skinned membrane with 0.05 CNT exhibited excellent normalized water flux of 81.4% after three successive fouling and cleaning cycles without sacrificing solute rejection. Such a performance demonstrated a bottom-skinned dense layer, which was effective in mitigating fouling by preventing the foulants from clogging the substrate pores.

Previously developed double-skinned TFC-FO membranes showed significant enhancement of antifouling property since dense PA was formed on the backside of the support, blocking the penetration of foulants into the membrane. Unfortunately, high water transport resistance from the second PA layer of double-skinned membrane could cause dramatic water fluxes with exceptionally lower water than the conventional TFC-FO membranes. Yang et al. [188] fabricated novel double-skinned FO membrane through an interfacial polymerization process. The double-skinned FO membrane comprising a hydrophilic nylon membrane filter substrate layer sandwiched between different selective skin layers: (i) a skin containing NaCl (0.15, 0.30, 0.45, 0.60 M) added into MPD, (ii) a dense skin without NaCl. The reserved NaCl changed the effective driving force between the active layer, which improved the water flux by 14.58% for double-skinned FO membrane immersed in a 0.45 M NaCl compared to the typical single-skinned and double-skinned FO membrane. The reason was attributed to similar salt of substrate and draw solution, resulting in maximum osmotic pressure on both sides of the active layer. In addition, the ICP was suppressed as concentration gradient was reduced, implying a dense active layer on both sides of the substrate, preventing water from being taken out the reserved solute in the substrate. Simultaneously, the membrane also offered better fouling tolerance over the single-skinned membrane over long-term operation. The experimental results emphasized the significant part of the second skin layer in reducing the membrane fouling propensity by inhibiting the foulants entering the substrate, leaving only accumulated foulants on the membrane surface. Almost all foulants were separated by means of simple physical flushing, leading to a more cost-effective and robust FO operation.

**Figure 9:**
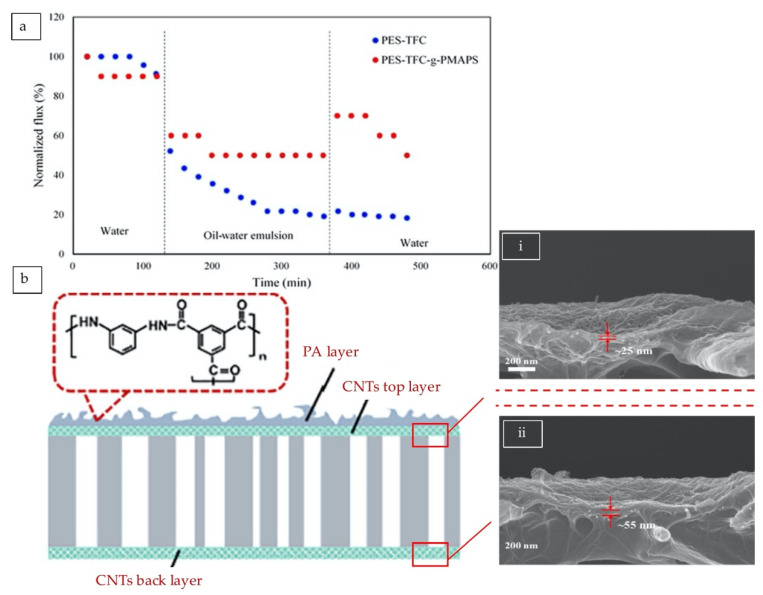
(**a**) Water flux recovery of single-skinned and double-skinned TFC membranes over a period of 480 min [189]. (**b**) Schematic of sandwich-like CNT-PES-CNT with cross-sectional SEM images of (i) CNT top layer and (ii) CNT back layer [31].

Ong et al. [189] delivered a significantly enhanced fouling resistance of FO membrane by fabricating double-skinned TFC membrane for emulsified oil–water separation (oily wastewater treatment). The top dense PA layer on PES substrate was synthesized via interfacial polymerization, meanwhile the bottom layer was zwitterionic copolymer layer made up of a zwitterionic brush, poly(3-(-2-methacryloxyethyl-N,N-dimethyl) ammonatopropane-sultone) (PMAPS). The double-skinned membrane had somewhat superior hydrophilicity with fully porous sublayer sandwiched between a highly dense PA layer and a fairly loose dense bottom zwitterionic layer, which resulted in maintaining high salt rejection and oil particle removal. The quality of permeation water from oily wastewater meliorated (purity > 99.9%) at a reasonably good water flux of 13.7 ± 0.3 L m^−2^h^−1^ using 2 M NaCl as the draw solution and oily solution as feed. From Figure 9a, recovery water flux significantly reduced over time due to internal (oil particles entered the porous PES) and external fouling (oil particles deposited on the membrane surface). Comparing both membranes, the resultant double-skinned membrane showed higher water flux recovery of 70%, threefold higher than that of the single-skinned membrane. This can be explained by the superior hydrophilic properties of the zwitterionic layer, which protected emulsified oil particles from fouling faster on the TFC surface, hence reducing internal fouling and ICP effects to some extent.

Zhang et al. [41] utilized polyketone as a substrate to fabricate double-skinned FO membrane for wastewater application. Double-skinned membrane consists of PA layer (salt-rejecting) on the top and a zwitterionic brush, poly(sulfobetaine methacrylate) (PSBMA)-decorated MWCNT (MWCNT/PSBMA) as foulant-resisting layer on the back side. The former top skin is a polyamide synthesized via interfacial polymerization, while the latter is constructed by a vacuum filtration coating method. Despite strong hydrophilicity and excellent foulant resistance of the zwitterionic brush, its small sizes cannot fully cover such large pores on the membrane back side. Hence, the zwitterionic brush was grafted on the MWCNT before coating the porous polyketone substrate, owing to its promising properties. Results showed less foulant adhesion (alginate gel) and no internal fouling in comparison with the neat forms as MWCNT/PSBMA successfully defensed them from entering the pores. Coating an MWCNT/PSBMA layer on the substrate achieved the highest flux retention up to 82% of its initial value, demonstrating the outstanding antifouling properties derived from the MWCNT/PSBMA surface.

Deng et al. [31] designed a new FO membrane to achieve high flux and high power density when operated in the AL-DS orientation. Sandwich-like SWCNT-coated substrate was prepared by coating PDA-modified SWCNTs on both sides of the PES substrate for high flux and antifouling TFC-FO/PRO membranes. CNT top and back layer of substrate were generated by different coating techniques: vacuum-filtration, and auto-spraying certain amounts of PDA-SWCNT dispersion on the PES, respectively. Morphological characterization in Figure 9b shows the SWCNT layer tightly attached on both surfaces of the PES membrane while the SWCNT top layer was thinner, looser, and smoother compared to the SWCNT back layer. Thanks to the construction of the smooth SWCNT top layer, the formation of the thin and small pore PA layer was able to perform better in terms of improved FO permeability and superior antifouling properties. Correspondingly, CNT-PES-CNT membrane showed a superior perm-selectivity with an increased water flux by ≈18.5% and a reduced reverse salt flux by ≈32.1%. Meanwhile, the back layer obstructed the foulant adsorption and intrusion into the PES support. This sandwich-like SWCNT-coated PES exhibited excellent antifouling properties with superior BSA rejection up to 98.1% and low relative fouling degree of 19.0% during the crossflow run. Beneficially lower flux decline was observed compared to the neat membrane.

## 6. Perspectives and Conclusions

In this membrane technology, the long-held goal is to design membranes with the highest permeate flux and solute rejection while keeping the costs and energy consumption of the membrane production to a minimum in order to be industrially established at a large scale. Therefore, it is something that entails researchers’ immediate action that should be well documented. The exponential increase of publications and patterns on this technology have witnessed the fundamental understanding of and major challenges in ICP and fouling modelling of FO membrane according to the growth of research activities in many applications, particularly wastewater treatment and desalination. This review is an attempt to provide a basis for the rational selection and modification protocols of the substrate layer on the basis of previous works, inasmuch as the literature has pointed out that a wide range of physical and chemical strategies have been explored on the substrate layer in achieving a favorable membrane with respect to structural properties and performance. Table 1 and Table 2 summarize the strategies performed upon FO substrates presented in this review and their associated performance for desalination and wastewater treatment, respectively. Most of these modifications have successfully shown superiority in implementing hydrophilicity, functionality, selectivity, long-term durability, and antifouling nature to eliminate the intrinsic bottleneck ICP and fouling problem. Currently, the blending method is the most commonly practiced strategy of modifying FO membrane substrate ascribed to its relatively easy operation.

Above all, the advancement of novel FO membranes based on new eco-friendly materials and effective chemical modifications make the FO process a more promising alternative for various applications. In terms of material selection, we anticipate more attention being paid to exploring the use of block copolymer porous substrates that have outstanding features such as uniform pore size on the surface, less tortuosity, better pore interconnectivity, and high porosity when compared to common PES and PSf substrates. Improvement on the porosity and pores (introducing inter-connected pores) are beneficial in terms of providing additional flow paths for the water to easily wash out the solute and hence offset the restrictions brought about by the conventional substrate layer structure. Importantly, the stability and uniformity of modifications across a large membrane area together with chemicals used must be addressed in developing a versatile FO membrane with advanced performance and a sustainable and reliable manufacturing processes in order to possibly open up the value chains and realize their full economic potentials, especially for provision of both clean water and energy. Hence, enhancing the advancement of TFC-FO membranes remains exceedingly desirable and requires thorough study. In the era of water scarcity, nanotechnology has expanded rapidly across virtually all industrial sectors and academia in relation to water treatment membranes due to several key advantages that conventional manufacturers cannot offer such as enabling new functionality. Aiming to prolong the membrane lifetime as well as separation performance, an improvement from single nanofiller- to multi-nanofiller-incorporated substrate proved to further elevate water permeation and rejection rate performance, mostly in NF and RO. For instance, nanocomposite of TiO_2_ and CNTs in casting solution results in pleasant compatibility and favorable hydrophilicity substrate [192]. Reinforced mechanical properties accompanied by strong antifouling properties induced by the nanocomposite such as synthesized membrane would promote better separation performance, i.e., water permeation and salt retention. Therefore, such a combination of unique merits from the different nanofillers should be well explored for FO applications. Detailed valuation of the stability of the nanocomposite membrane are needed for controlling nanomaterial leaching in order to maintain durability. It is worthwhile to have an advanced fundamental understanding on the transport phenomena across nanocomposite substrate for the in-depth utilization nanocomposite FO membrane to improve its performance and properties. As a drive to produce more water resources from wastewater, an extensive research and analysis is still needed for fouling mechanisms caused by substrates to identify effective antifouling strategies in preparing improved productivity and durable antifouling membranes. This will benefit the optimization of membrane design for desalination and wastewater treatment purposes. Less attention has been paid to the blending and subsequent inclusion of mixed additives such as pore-forming PVP mixed with PEG, for which their potential combined effects may work under the appropriate blending to form a substrate with dense top surface and porous bottom surface so that the trade-off relationship between the selectivity and permeability could be minimized.

While the surface modification of the membranes using nanofillers has been shown to retain high water permeance, multistep procedures should be avoided in order to achieve higher reproducibility. Applying high-speed coating methods to redesign membrane with coating layer, i.e., interlayer, LbL, and double-skinned membrane may overcome the difficulties. Meanwhile, inclusion of crosslinking in coating technique together with understanding the structure–property relationships of coating materials may increase the mechanical integrity and membrane durability of coating layer (non-covalently bonded to substrate) that usually leaches from the surface overtime. Moreover, exploiting electrospinning to produce nanofibers with promising characteristics is a particularly effective approach in FO. Nevertheless, significant room for improvement exists in terms of further enhancing the mechanical integrity of the fiber mats. More studies are expected with regards to the post-treatments or precursor materials and parameters of electrospun nanofibrous substrates. Moreover, the prospect of developing antifouling electrospun membrane with a low *S* value is exciting in terms of achieving flux beyond the theoretical capacity. Therefore, it would be worthwhile to investigate the effects of the of substrate properties on the adhesion between the PA layer and nanofibrous substrates. Despite the available modification, converging suitable nanomaterials with nanofiber could offer better opportunities in preventing the growth of fouling layers on membrane surfaces.

In fact nanofillers contribute to the development of novel nanocomposite membranes, and researchers are concerned about their extensive use in relation to sustainability and potential risk, i.e., reactivity, persistence, and human safety [193]. Proper environmental safety and health management system should be established by employing quality check and risk evaluation protocols to guarantee a safe adoption and positive acceptance of thin film nanocomposite toward the next generation of the nano-structure membrane technologies. In addition, avoiding chemical by-products, excellent control, and response to process changes would drive membrane innovation and application expansion. Alternatively, this can be realized by the development of new smart FO membranes could be a new solution for efficient water treatment. Janus membranes with asymmetric properties on both sides appear as a kind of efficient membrane for the separation of oil in water emulsion. The latest finding by Zhou et al. reported on unparallel performance for FO using Janus membrane fabricated using PVDF and cellulosic membrane via electrospinning [194]. Such high flux and selectivity obtained can be originated from the Janus property of the composite membrane, i.e., a hydrophilic cellulose acetate layer depositing on a mechanically stable hydrophobic PVDF layer serving as a blocking barrier for reverse ion diffusion. Since literature data on Janus membranes for FO is limited, further exploration and optimization of Janus membranes may therefore find potential application in osmotically driven applications such as desalination and wastewater treatment.

As suggested by theoretical studies, implantation of FO membrane technology contributes up to 50% energy benefit, which can be a sustainable solution for upgrading a conventional water treatment plant [195,196]. To ascertain the viability and corroborate the potential cost, pilot scale studies have been implemented to provide a more practical guideline for FO system design and installation design. A pilot scale study has indicated that FO is also capable of achieving high water recovery (70%) for wastewater, even after 24 h using Aquaporin biomimetic membranes [197]. The value was higher than that achieved by a typical RO process (35–40%). Pilot-scale performance of the concentration of skim milk and whey using CTA-FO membrane proved that by employing the FO process, specific energy required is much lower (5–10 kWh/t water removed) than that required by the RO process. This demonstrates that FO is an effective technology for dairy applications if there is an access to a brine stream as a draw stream. Another pilot study combined FO pretreatment and RO desalination hybrid system as an alternative to process feed waters that cannot be treated by RO. Ali et al. [198] cotreated seawater and impaired water from the steel industry, wherein FO produced good results in seawater dilution, reaching a 250% dilution rate using the FO system. However, water flux in the FO process was found to decrease slightly by time due to the fouling.

However, the real-in field applications of TFC-FO membranes for water treatment is still in its infancy. To facilitate knowledge transfer, more pilot studies on FO systems relying on more robust practical and large-scale operations should be established and studied with continuing monitoring and analysis. More pilot-scale studies in this direction are desired in order to look into possible hiccups and aspects to be improved before the deployment of FO for large-scale commercial applications. Such up-scaling studies should be performed on a case-by-case basis with a full consideration of the source water quality and application environments. Conducting more research in these areas through focusing on membrane replacement costs and reducing pretreatment requirement should be possible in order to establish FO as a treatment technology in manufacturing industries.

## Figures and Tables

**Table 1:** Overview of the efficiency of different substrate modifications for FO desalination application.

Substrate	Modifying Agent	Rejection (%)		Flux (LMH)		S-Parameter (µm)		Reference
		Before	After	AL-FS	AL-DS	AL-FS	AL-DS	
**Electrospun Nanofibers**								
PSf nanofiber	PAN	95.35	97.12	-	38.3	340	-	[51]
PAN nanofiber	Membrane alignment	93.2	90.3	50.7	62.9	86.4	-	[150]
**Sulfonated Polymer Blending**								
PSf flat sheet	Sulfonated PSf 45 (16% sulfonation degree)	-	-	39.00	69.44	114.0		[85]
PES flat sheet	Sulfonated PES (50% sulfonation degree)	93.2	91.1	35.1	42.1	245.0		[155]
PSf flat sheet	2.5 wt % sulfonated PSf (20% sulfonation degree)	96.01	95.12	25.53	48.37	337.0		[153]
PVDF flat sheet	3 wt % perfluoro-sulfonic acid	15.18	92.23	27.0	54.4	334.6		[64]
PSf flat sheet	25 wt % disulfonated poly (arylene ether sulfone) multiblock copolymer	99.41	98.95	40.9	74.4	186.0	397.0	[120]
**Pore Former Blending**								
PSf flat sheet	PEG-400	97.0	96.5	47.4	-			[125]
PSf flat sheet	PSf-mPEG500	96.0	96.0	16.50				[190]
CAT flat sheet	2 wt % PVB	90.7	86.6	16.8	27.5			[156]
PSf flat sheet, PES flat sheet, PSf flat sheet	3 wt % LiCl	94.3	95.3 94.8 97.6	6.71 6.88 5.72	7.29 10.67 8.48			[65]
**Nanofiller Blending**								
PES flat sheet	MWCNT	90.32	90.68	11.98	-			[147]
PVDF flat sheet	SiO_2_@MWNTs	78.2	96.3	22.1	29.5			[168]
CTA flat sheet	0.5 wt % CNF	80.0	84.0	15.6	15.8			[29]
PSf flat sheet	0.33% INT	58.0	83.0	8.0	10.0			[191]
PSf flat sheet	0.5% protonated carbon nitride	98.0	94.5	4.24	11.0			[169]
PSf flat sheet	0.5 wt % TiO_2_/GO	96.0	94.4	24.0	30.0			[137]
PES flat sheet	0.2 wt % Fe_3_O_4_	96.3	93.2	17.5	21.9			[134]
PES flat sheet; PEI flat sheet	MOF-5	97.47	97.37	32.74; 21.78	-			[175]
**Template-Assisted**								
PSf flat sheet	7.5 wt % LDH-HCl	93.8	92.4	20.1	47.2	-	-	[180]
PSf flat sheet	5 wt % SiO_2_-HF	97.1	94.5	60.8	≈70.0	132.28	-	[176]
**Hydrophilic Coating**								
PE flat sheet	PDA	98.1	97.4	40.7	43.2			[181]
PES flat sheet	PAA5/CaCO_3_	>90	>90	52.0	62.0			[96]
PSf flat sheet	PDA/ 0.5 GO interlayer	92.8	98.0	24.30	-			[147]
PES flat sheet	CNT interlayer	-	-	-	38.0			[182]
**Layer-by-Layer and Double-Skinned Membrane**								
PES flat sheet	Crosslinked PEI-PAA LbL	-	-	22.48	-	-	-	[45]
PES flat sheet	GO-CNTs LbL	-	70.2	9.0	-	-	-	[186]
PSf flat sheet	Double-skinned 0.05 wt % CNT	-	-	8.8	12.4			[187]

**Table 2:** Performance and modification comparison of substrate modifications for wastewater treatment

Substrate	Modifying Method/Agent	Application	Retention (%)	Flux (LMH)	Reference
				FO mode	
PSf nanofiber	Electrospinning	Antibiotic wastewater	Antibiotics: 98.1–99.7	48.98	[43]
PSf flat sheet	5 % SPEEK	High salinity wastewater	NaCl: 96.1	28.3	[160]
PSf flat sheet	4% PEG 400	Synthetic, industrial wastewater	Pb^2+^, Cd^2+^, Cr^2+^: 99.9, 99.7, 98.3	34.4	[159]
20.4 wt % PESU 18 wt % Matrimid 15 wt % sPPSU	-	Organic pollutant wastewater	Aniline, phenol, nitrobenzene 91.8, 73.7, 76.8 88.4, 75.9, 76.6 91.5, 72.1, 75.2	20.6, 14.1, 22.1	[161]
SPES flat sheet	PVP PEG glycerol	Domestic wastewater	NH_4_^+^, PO_4_^3-^, TOC: 56.31, 93.7, 93.01 59.06, 98.1, 97.01 58.22, 96.0, 97.48	38.18 35.08 33.58	[79]
PES flat sheet	Double-skinned/ zwitterionic brush	Oily wastewater	Oil: 99.0	13.7	[189]
PES flat sheet	Coating/PDA/SWCNT interlayer	Salinity gradient recovery	BSA: 98.0	18.1	[31]
